# Next generation sequencing‐based copy number analysis reveals low prevalence of deletions and duplications in 46 genes associated with genetic cardiomyopathies

**DOI:** 10.1002/mgg3.187

**Published:** 2015-12-16

**Authors:** Ozge Ceyhan‐Birsoy, Trevor J. Pugh, Mark J. Bowser, Elizabeth Hynes, Ashley L. Frisella, Lisa M. Mahanta, Matt S. Lebo, Sami S. Amr, Birgit H. Funke

**Affiliations:** ^1^ Laboratory for Molecular MedicinePartners HealthCare Personalized MedicineCambridgeMassachusetts; ^2^ Department of PathologyBrigham and Women's Hospital and Harvard Medical SchoolBostonMassachusetts; ^3^ Department of Medical BiophysicsPrincess Margaret Cancer CentreUniversity Health NetworkUniversity of TorontoTorontoOntarioCanada; ^4^ Department of PathologyMassachusetts General Hospital and Harvard Medical SchoolBostonMassachusetts

**Keywords:** Cardiomyopathies, DNA copy number variants, genetic heterogeneity, molecular diagnostics, next‐generation sequencing

## Abstract

**Background:**

Diagnostic testing for genetic cardiomyopathies has undergone dramatic changes in the last decade with next generation sequencing (NGS) expanding the number of genes that can be interrogated simultaneously. Exon resolution copy number analysis is increasingly incorporated into routine diagnostic testing via cytogenomic arrays and more recently via NGS. While NGS is an attractive option for laboratories that have no access to array platforms, its higher false positive rate requires weighing the added cost incurred by orthogonal confirmation against the magnitude of the increase in diagnostic yield. Although copy number variants (CNVs) have been reported in various cardiomyopathy genes, their contribution has not been systematically studied.

**Methods:**

We performed single exon resolution NGS‐based deletion/duplication analysis for up to 46 cardiomyopathy genes in >1400 individuals with cardiomyopathies including HCM, DCM, ARVC, RCM, and LVNC.

**Results and Conclusion:**

Clinically significant deletions and duplications were identified in only 9 of 1425 (0.63%) individuals. The majority of those (6/9) represented intragenic events. We conclude that the added benefit of exon level deletion/duplication analysis is low for currently known cardiomyopathy genes and may not outweigh the increased cost and complexity of incorporating it into routine diagnostic testing for these disorders.

## Introduction

Truly comprehensive molecular testing for diseases with high genetic heterogeneity and clinical variability is challenging for diagnostic laboratories as it requires capturing a wide variant spectrum in a large number of genes. The adoption of next‐generation sequencing (NGS) has transformed the testing approach for these disorders by expanding the number of genes that can be targeted in a time‐ and cost‐effective manner. However, until recently NGS, like its predecessor Sanger sequencing, was largely restricted to detecting single nucleotide variants and small insertions/deletions. Despite the fact that CNVs are well‐recognized causes of a wide range of genetic disorders (reviewed in (Zhang et al. 2009) and (Stankiewicz and Lupski 2010)), most molecular diagnostic laboratories have traditionally not included comprehensive copy number assessment for their gene panels due to the high cost and impracticality of scaling conventional molecular diagnostic CNV detection methods such as MLPA and qPCR. On the other hand, genome‐wide CNV testing is already routinely performed by cytogenomic laboratories, but has usually been focused on very large deletions and duplications. One consequence of this historical separation of technologies and approaches is a largely unexplored class of small, intragenic single or multi exons deletions and duplications. For simplicity, the term CNV is used throughout this manuscript to include intragenic events although these do not represent copy number alterations in a traditional sense.

Two recent developments promise to enable a combined analysis of small sequence variants as well as CNVs. Improved high‐resolution cytogenomic arrays allow exon level CNV detection and are being used to supplement NGS gene panel or exome sequencing in laboratories proficient in both methodologies. An emerging alternative strategy is NGS‐based CNV detection, which has the potential of consolidating assays needed for comprehensive variant detection and therefore streamlining diagnostic testing (Zhao et al. 2013; Retterer et al. 2014). Emerging data show that overall, intragenic deletions or duplications constitute an important portion of the so‐called missing variation. However, currently available NGS‐based detection methods are all afflicted by a high false positive rate, especially for small intragenic deletions and duplications, which increases the cost associated with orthogonal confirmation (Feng et al. 2015).

The addition of comprehensive deletion/duplication testing to traditional sequence analysis has significantly improved the diagnostic yield for some disorders, including hearing loss and retinal dystrophies (Eisenberger et al. 2013; Shearer et al. 2014) but this has not been systematically evaluated for many other disorders.

For inherited cardiomyopathies, it is now common practice to sequence large gene panels including at least 20 genes (Teekakirikul et al. 2013; Pugh et al. 2014). Several genes are known to be susceptible to haploinsufficiency based on the high prevalence of heterozygous loss of function variants. Prominent examples include *MYBPC3*, which explains 15–20% of hypertrophic cardiomyopathy (HCM) and *TTN*, which is responsible for 10–25% of dilated cardiomyopathy (DCM) (Herman et al. 2012; Teekakirikul et al. 2013; Pugh et al. 2014; Alfares et al. 2015). A contribution of copy number variation would be plausible and has been shown to exist for some cardiomyopathy genes. For example, deletion of single or multiple exons of *LMNA* and *BAG3*,* MYBPC3*, and *PKP2* have been reported in individuals with dilated, hypertrophic (ARVC), or arrhythmogenic right ventricular cardiomyopathy, respectively (Jouven et al. 2002; Gupta et al. 2010; Kapplinger et al. 2011; Norton et al. 2011; Chanavat et al. 2012; Groeneweg et al. 2013; Li Mura et al. 2013), but the overall prevalence of CNVs in cardiomyopathy‐associated genes remains incompletely characterized.

Because implementation of comprehensive clinical copy number testing for large gene panels requires consideration of many factors including technical platforms, their analytical performance and associated orthogonal confirmation cost, it is essential to understand whether the resulting increase in diagnostic yield justifies the increased cost associated with added CNV testing. Our work represents one of the first systematic evaluations of the prevalence of CNVs in individuals with inherited cardiomyopathies, which can help guide decisions on whether and how to implement CNV testing for these disorders.

## Materials and Methods

### Patient cohort and next‐generation sequencing

The patient cohort included in this study represents a broad worldwide referral population of 1425 individuals with reported clinical diagnosis of HCM (*n* = 708), DCM (*n* = 479), ARVC (*n* = 90), left ventricular noncompaction (LVNC) (*n* = 54), restrictive cardiomyopathy (RCM) (*n* = 25), cardiomyopathy not otherwise specified (NOS) or a combination of different cardiomyopathy features (*n* = 61), or cardiomyopathy accompanied by skeletal myopathy features (*n* = 8), as described on their test requisition. Detailed clinical data to independently verify the diagnoses was not available (see Limitations section below). Testing was done at the Laboratory for Molecular Medicine of Partners HealthCare Personalized Medicine. This study was approved by the Partners HealthCare Institutional Review Board. The number of genes tested varied: 795 individuals received comprehensive testing of 46 genes covering the full spectrum of cardiomyopathies (Table S1). An additional 630 patients received disease‐specific testing as requested by their healthcare provider using the following gene panels: HCM panel (18 genes, 457 individuals), DCM panel (24 genes, 109 individuals), ARVC panel (8 genes, 46 individuals), and LVNC panel (10 genes, 18 individuals) (Tables S1 and S2). This cohort was predominantly White (825 were White, 123 were Black or African American, 43 were Hispanic or Latino, 55 were Asian, 34 were Ashkenazi Jewish, 4 were American Indian or Alaska Native, 3 were Native Hawaiian, or Other Pacific Islander, 45 were of mixed ancestry, and 293 were of unspecified ancestry). NGS, confirmation, and interpretation of single nucleotide variants (SNVs) were performed as described (Duzkale et al. 2013; Pugh et al. 2014).

### Deletion/duplication detection from ngs data

A custom R‐based tool (VisCap), which compares the fractional coverage of each exon to the median of these values across all samples in a sequencing run (10 samples per batch), was used for CNV detection (Pugh et al. in press). Briefly, coverage across each interval captured was calculated using the “DepthOfCoverage” program of the Genome Analysis Toolkit (GATK) (McKenna et al. 2010). Fractional coverage values were compared by dividing each value by the median for that target across the entire batch. CNVs were called using fixed thresholds representing the minimum log2 ratio for gains (0.40) and maximum log2 ratio for losses (−0.55). The final output of the tool are log2 ratios plotted by relative genome order (see Fig. S1 for representative VisCap visual outputs). The full code is available upon request. The sensitivity of this approach was 100% for both copy number losses and gains detection (Pugh et al., in press). In order to improve specificity in our study, we manually reviewed all VisCap output plots and employed a visual scoring system that enabled experienced reviewers to differentiate true positive calls from false positives, which increased the specificity from 37 to 87% (Pugh et al., in press).

### Orthogonal confirmation of deletions and duplications using droplet digital PCR

Deletions and duplications with potential clinical significance (as described in Results) were validated using droplet digital PCR (ddPCR) (Hindson et al. 2011). Calls made in ≥1% of samples were not further analyzed (Table S3). Briefly, DNA samples were fragmented with MseI enzyme (New England Biolabs, Ipswich, MA). TaqMan assay pools were prepared using 2X ddPCR Master Mix (BioRad, Waltham, MA), 20X target assay consisting of target primers (IDT) and FAM‐labeled TaqMan target probes (Life Technologies, Waltham, MA), 20X reference assay consisting of reference primers and VIC‐labeled TaqMan probes for the *RPP30* gene, and DNA template in a final volume of 25 *μ*L. Reaction mixtures were transferred and droplet generation oil (BioRad) was added into the sample column of a droplet generator (BioRad) to create droplets. Collected droplets were transferred into a 96‐well PCR plate and amplified to the end‐point (40 cycles) on a thermocycler. Automated analysis of the data output was performed using QuantaSoft analysis software (BioRad).

## Results

### Detection and clinical classification of CNVs

CNVs identified by NGS were investigated via visual assessment and confirmed by ddPCR as described above. Variants were classified in line with the American College of Medical Genetics and Genomics and the Association for Molecular Pathology (ACMG and AMP) standards and guidelines for the interpretation of sequence variants (Richards et al. 2015), by assessing the frequency in patient and control populations, de novo occurrence or segregation with disease, functional evidence, predicted protein impact, and the spectrum of pathogenic variation in a gene. Nine CNVs were classified as either pathogenic, likely pathogenic, or variant of uncertain significance (VUS) and are summarized in Table 1.

**Table 1:** Clinically significant copy number variants identified in cardiomyopathy patients

Patient ID	Sex	Age	Diagnosis	Additional cardiac features	Family history	Gene	Region	Loss/gain	Classification	Other variants	Test
51	M	36 years	DCM	VT	DCM	LMNA	Exon 1	Loss	Likely pathogenic	RYR2 c.2755G>A (p.Val919Met) (Het),VUS TTN c.98417T>A (p.Phe32806Tyr) (Het),VUS TTN c.41249T>C (p.Ile13750Thr) (Het), VUS	PanCardio panel
450	M	49 years	DCM		DCM, SCD	TTN	Exon 193–244	Gain	VUS	VCL c.1844C>T (p.Ala615Val) (Het), VUS	DCM panel
1060	F	39 years	DCM	WPW, glycogen noted at transplant	DCM	LAMP2	Exon 8–9	Loss	Pathogenic	TTN c.38361G>C (p.Lys12787Asn) (Het),VUS	PanCardio panel
104	M	55 years	HCM		Unknown	MYOZ2	Exon 2	Gain	VUS	None	HCM panel
267	F	44 years	HCM		HCM	MYBPC3	Exon 12–20	Loss	Pathogenic	None	HCM panel
958	M	57 years	HCM		HCM, SCD	NEXN	Whole gene	Gain	VUS	MYH7 c.1063G>A (p.Ala355Thr) (Het), VUS PKP2 c.2365A>G (p.Ile789Val) (Het), VUS TMPO c.1174C>T (p.Pro392Ser) (Het), VUS TTN c.22796C>T (p.Thr7599Met) (Het), VUS	PanCardio panel
1044	F	1 month	HCM	Multiple VSDs	Unknown	GLA/LAMP2/EMD/TAZ	X chr	Gain	VUS	LDB3 c.733G>A (p.Val245Ile) (Het), VUS SCN5A c.659C>T (p.Thr220Ile) (Het), VUS	PanCardio panel
322	M	1 year	LVNC		Unknown	PKP2	Whole gene	Gain	VUS	TTN c.29071A>G (p.Lys9691Glu) (Het), VUS	PanCardio panel
1233	M	15 years	ARVC	Reduced LV function	Abnormal signal averages on ECG	PKP2	Exon 8	Loss	Likely pathogenic	TTN c.15284A>G (p.Tyr5095Cys) (Het), VUS TTN c.60547A>C (p.Asn20183His), (Het), VUS TTN c.11687T>A (p.Ile3896Asn) (Het), VUS TTN c.13163T>C (p.Ile4388Thr) (Het), VUS TTN c.24455C>T (p.Pro8152Leu) (Het) VUS	PanCardio panel

VT, ventricular tachycardia; VSD, ventricular septal defect; LV, left ventricle; WPW, Wolff Parkinson White; SCD, sudden cardiac death; VUS, variant of uncertain significance.

### Overall contribution of CNVs to cardiomyopathies

Overall, clinically relevant CNVs were identified in 9 of 1425 (0.63%) individuals with cardiomyopathies. Four of these variants were classified as pathogenic or likely pathogenic (0.28%). Four variants were intragenic deletions (in *LMNA*,* LAMP2*,* MYBPC3*, and *PKP2*), two were intragenic duplications (in *TTN* and *MYOZ2*), and two were whole gene duplications (*NEXN* and *PKP2*). We identified a trisomy X that was detected due to the duplication of four genes tested on the X chromosome (*GLA*,* LAMP2*,* EMD*, and *TAZ*). Table 2 lists the detection rate per gene. *PKP2* and *LAMP2* were the only two genes with more than one CNV identified in our patient cohort (2/850 and 2/1361 tested, respectively). The vast majority of genes (36/46) did not have any clinically significant CNVs in ≥795 patients tested (Table 2). A clinically significant CNV was identified in 1.9% (1/54) of LVNC, 1% (1/99) of ARVC, 0.6% (3/479) of DCM, and 0.4% (4/708) of HCM patients. No clinically significant CNVs were identified in patients with RCM or cardiomyopathy NOS.

**Table 2:** CNV detection rate per gene and predicted outcomes**.**

A. Genes with CNVs identified in cardiomyopathy patients					
Gene	Number of patients tested	Number of patients with clinically significant CNVs	CNV type	Predicted outcome of CNV	Known disease mechanism of variants in gene for cardiomyopathy
*EMD*	904	1	Whole gene dup	Unknown	LOF
*GLA*	1252	1	Whole gene dup	Unknown	LOF
*LAMP2*	1361	2	Whole gene dup, Intragenic del	Unknown, LOF	LOF
*LMNA*	922	1	Intragenic del	LOF	LOF
*MYBPC3*	1379	1	Intragenic del	LOF	LOF
*MYOZ2*	1252	1	Intragenic dup	LOF	Unknown
*NEXN*	1361	1	Whole gene dup	Unknown	Unknown
*PKP2*	841	2	Whole gene dup, Intragenic del	LOF, LOF	LOF
*TAZ*	922	1	Whole gene dup	Unknown	LOF
*TTN*	904	1	Intragenic dup	LOF	LOF

B. Genes with no CNVs identified in cardiomyopathy patients			
Gene	Number of patients tested	Gene	Number of patients tested
*ABCC9*	904	*MYH6*	795
*ACTC1*	1379	*MYH7*	1379
*ACTN2*	1361	*MYL2*	1252
*ANKRD1*	795	*MYL3*	1252
*CASQ2*	859	*MYLK2*	795
*CAV3*	795	*PLN*	1361
*CRYAB*	795	*PRKAG2*	1252
*CSRP3*	1361	*RBM20*	904
*CTF1*	904	*RYR2*	841
*DES*	904	*SGCD*	904
*DSC2*	841	*TCAP*	904
*DSG2*	841	*TMEM43*	841
*DSP*	841	*TNNC1*	1361
*DTNA*	813	*TNNI3*	1361
*FHL2*	795	*TNNT2*	1379
*JUP*	841	*TPM1*	1361
*LAMA4*	795	*TTR*	1252
*LDB3*	922	*VCL*	922

dup, duplication; del, deletion; LOF, loss of function; GOF, gain of function.

### Clinically significant intragenic deletions

Four intragenic deletions (in *LAMP2, LMNA, MYBPC3,* and *PKP2)* were predicted to lead to truncated or absent proteins and were classified as pathogenic or likely pathogenic, since the pathogenic variant spectrum of these genes is known to contain a significant fraction of loss of function variants (Pugh et al. 2014; Alfares et al. 2015). The reported clinical diagnoses for the four patients with these variants were consistent with the respective results (Table 1 and below).

#### *LAMP2*

Loss of function of this gene is associated with Danon disease, an X‐linked glycogen storage disorder characterized by cardiomyopathy, arrhythmias, skeletal myopathy, and intellectual disability (Boucek et al. 2011). HCM is the predominant cardiomyopathy in males. Although heterozygous females are often asymptomatic, they may present with later‐onset cardiomyopathy, most often DCM, and masquerade as nonsyndromic since additional features leading to a suspicion of Danon disease may not be present. Large deletions in *LAMP2* have been previously described in seven patients with Danon disease (Lacoste‐Collin et al. 2002; Fanin et al. 2006; Yang et al. 2010; Boucek et al. 2011). Here, we identified an intragenic deletion in *LAMP2* in a female patient with DCM, arrhythmia, and glycogen accumulation noted during cardiac transplant, consistent with Danon disease.

#### *LMNA, MYBPC3,* and *PKP2*

Loss of function variants are part of the spectrum of pathogenic variation for DCM (*LMNA*), HCM (*MYBPC3*), and ARVC (*PKP2*), which is consistent with the reported clinical diagnoses of the patients carrying these variants. Variants causing loss of function of the *MYBPC3* gene, many of which are frameshift, nonsense, or splice variants, explain ~16% of HCM cases (Alfares et al. 2015). Similarly, SNVs and small indels in *PKP2* gene that abolish or impair protein function account for 20–46% of ARVC cases (den Haan et al. 2009; Teekakirikul et al. 2013; Groeneweg et al. 2015; Lazzarini et al. 2015). Interestingly, we identified only a single HCM patient with a large deletion in *MYBPC3* in 708 HCM patients (0.14%), and one *PKP2* exon 8 deletion in 90 ARVC patients (1.1%) that was confirmed to segregate with disease in an affected family member. Additionally, although variants leading to loss of function of *LMNA* explain ~4% of DCM (Pugh et al. 2014), we identified only one individual with *LMNA* exon 1 deletion in 479 DCM patients (0.2%).

### Clinically significant duplications

#### Whole gene duplications

We identified whole gene duplications of *NEXN* and *PKP2* in patients with HCM and LVNC, respectively. The spectrum of pathogenic variants in *NEXN* is still largely uncharacterized. The proposed molecular mechanism of pathogenicity is a dominant‐negative effect due to an altered nexillin interaction with actin or other sarcomere components, but this rests on only five missense or single amino acid deletion variants in individuals with HCM and DCM (Hassel et al. 2009; Wang et al. 2010). The variant spectrum of *PKP2* is far better characterized. More than 50% of disease‐causing variants described in *PKP2* are predicted to lead to loss of function in the protein (Lazzarini et al. 2015). Gain of function or increased copy number has not yet been described for either gene, and *PKP2* has not been associated with LVNC. Therefore, both of these whole gene duplications were classified as “variants of uncertain significance” (VUSs). Duplication of all four genes on our X‐chromosome target region was identified in one patient with clinical diagnosis of HCM (Fig. S1C). This case was confirmed to have trisomy X by karyotyping. Although congenital structural heart defects are known to be in the clinical spectrum of trisomy X (Haverty et al. 2004; Roth et al. 2006; Bagci et al. 2009), cardiomyopathy has not been previously reported in this disorder. Therefore, whether trisomy X contributes to the cardiomyopathy in this patient is uncertain.

#### Intragenic duplications

We detected intragenic duplications in *MYOZ2* in a patient with HCM and in *TTN* in a patient with DCM. The majority of intragenic duplications have been shown to represent tandem duplications, while only ~2–2.8% of clinically relevant duplications are found to be insertional translocations (Kang et al. 2010; Neill et al. 2011; Nowakowska et al. 2012; Newman et al. 2015). Therefore, the most likely functional consequence of an intragenic duplication is disruption of the reading frame, resulting in loss of function of the protein. The functional consequence of an insertional translocation would depend on the expression status and genomic location of the duplicated fragment. The variants in *MYOZ2* and *TTN* that were identified in our study are consistent with the clinical diagnoses of the patients they were detected in. However, although the most likely predicted outcome of these variants is loss of function of the protein, since our methodology is unable to detect whether they truly represent tandem duplications versus insertional translocations, the clinical significance of these variants remain uncertain.

## Discussion

The overall clinical sensitivity of targeted NGS panels for genetic cardiomyopathies is less than 50% depending on the specific diagnosis (Pugh et al. 2014; Alfares et al. 2015). Adoption of technologies that allow hypothesis‐free testing in molecular diagnosis of cardiomyopathies has led to increased clinical sensitivity for many disorders and broadened both the spectrum of disease causing variants and the range of phenotypes associated with some genes. One example for this phenomenon is the *GLA* gene (Adalsteinsdottir et al. 2014; Alfares et al. 2015), which was traditionally tested only in patients with clinical diagnosis of Fabry disease, a lysosomal storage disorder that involves acroparesthesias, angiokeratomas, proteinuria, and cardiomyopathy (Germain 2010). Inclusion of *GLA* in NGS panels revealed a larger than expected fraction of pathogenic *GLA* variants among individuals with apparently nonsyndromic HCM (Adalsteinsdottir et al. 2014; Alfares et al. 2015). Similarly, testing a larger range of genes led to a better understanding of the clinical overlap between different groups of cardiomyopathies and has been beneficial for ending the diagnostic odysseys faced by patients with ambiguous or nonspecific presentations. This benefit has probably been most pronounced for DCM, as it can be an end‐stage presentation of HCM in a minority of cases (Biagini et al. 2005) and a clinical overlap exists between DCM and ARVC (Sen‐Chowdhry et al. 2008; van der Zwaag et al. 2012). Accurate clinical diagnosis can be challenging and it has not been surprising that pathogenic variation in desmosomal genes contribute to a portion of disease‐causing variation in DCM patients (Elliott et al. 2010; Pugh et al. 2014). These efforts demonstrated that a broader testing strategy that targets a wide genetic spectrum is beneficial for achieving maximum clinical sensitivity. In addition to a higher number of genes, targeting a wider variant spectrum is expected to lead to increased yield, as has been demonstrated for inclusion of CNV testing for many genetic disorders (Eisenberger et al. 2013; Shearer et al. 2014). To understand whether CNV testing would increase the diagnostic yield for cardiomyopathies, we have broadened our testing approach by including CNV analysis in our NGS panel for cardiomyopathies. We observed that CNVs have a negligible contribution to genetic cardiomyopathies overall. Given the high sensitivity of our copy number caller (100% for both copy number gains and losses; Pugh et al., in press), it is unlikely for this low prevalence to be due to a limitation of our detection method's ability to capture these alterations. Although SNVs or small indels predicted to result in a loss of function are prevalent in several cardiomyopathy genes (such as *MYBPC3*,* LMNA*,* TTN*, and *PKP2*), and most CNVs (whole gene and intragenic deletions, and intragenic duplications, with the exception of whole gene duplications) are expected to cause loss of function of the protein, CNVs do not appear to have a large contribution to the pathogenic variant spectrum of cardiomyopathy genes. One possible explanation for the low rate of copy number variation in these genes may lie in their chromosomal sequence context, not rendering them prone to molecular mechanisms that introduce copy number alterations (low copy repeats, short interspersed nuclear elements, etc.).

The low rate of CNVs in cardiomyopathy genes has potential implications for clinical laboratories offering cardiomyopathy testing. Diagnostic laboratories need to be as comprehensive as possible for the pathogenic gene and variant spectrum associated with their targeted disease, while considering the cost and time for such testing. Currently, intragenic CNVs are not routinely investigated, therefore are largely being missed by both molecular diagnostic or cytogenomic laboratories. NGS‐based CNV detection offers the ability to consolidate two testing modalities that have traditionally been applied separately. While this approach can lead to increased diagnostic yield, one needs to keep its relatively high false positive rate (Feng et al. 2015) in mind, which necessitates confirmation by a secondary method before results are reported to patients. Therefore, the addition of comprehensive CNV analysis to disease‐focused gene panels needs to be evaluated in the context of the added benefit in comparison to the potential cost and time for confirmatory testing. Our results do not suggest that a particular phenotype is more likely to indicate a CNV analysis over the others; however the genes we have identified deletions or duplications in should be considered for CNV analysis when the clinical diagnosis is consistent. Although the overall rate of CNV detection is low, their identification can be critical for patients, particularly since the detection of at risk family members by molecular testing once the genetic cause of familial cardiomyopathy is known may prevent devastating outcomes. Therefore, CNV analysis should be considered in genes and diseases for which the clinical benefit of their detection would outweigh the potential added cost and turnaround time of testing.

### Limitations

Our study relied on clinical data provided by ordering physicians at the time of testing. In contrast to a controlled clinical study in which all patients are evaluated using a common set of diagnostic criteria, our clinical data are likely more heterogeneous.

CNV calls common in ≥1% of our cohort were excluded from analysis and were not tested by a secondary confirmatory method. We cannot exclude the possibility that true CNVs may lie in these regions; however, since their frequency would lead to a clinical classification of “likely benign” or “benign” they would not change the overall frequency of clinically significant CNVs in our cohort.

Our method only detects CNVs in our targeted capture region, which is limited to exonic regions of the genes on our NGS panel. Intronic or intragenic CNVs that do not cover exonic regions, but may have an impact on the expression or function of these genes cannot be ruled out.

Although the sensitivity of VisCap has been determined as 100%, this is based on a relatively small number of CNVs that were available to test during the validation of this tool, due to the rarity of these events in our patient populations.

There are currently no publicly available databases that are comprehensive for exon level deletion/duplication variants in the general population, therefore the existence of these variants in control individuals cannot be ruled out.

Finally, although our study is, to our knowledge, one of the largest reported analyses of CNVs in cardiomyopathy genes, it is not large enough to be immune to statistical fluctuations that commonly afflict small‐ to medium‐sized cohorts.

## Conflict of Interest

None declared.

## Supporting information


**Figure S1.** Representative VisCap visual outputs demonstrating (A) PKP2 exon 8 deletion, (B) RAF1 whole gene duplication, (C) Three copies of X chromosome.
**Table S1.** Gene content of next‐generation sequencing panels.
**Table S2.** Summary of patients included in the study.
**Table S3.** Frequency of CNVs called by VisCap.
