# Supplementary material for: Next generation sequencing‐based copy number analysis reveals low prevalence of deletions and duplications in 46 genes associated with genetic cardiomyopathies
Source: Mol Genet Genomic Med. 2015 Dec 16;4(2):143–51. doi: 10.1002/mgg3.187 (PMC4799872; doi:10.1002/mgg3.187)
Supplement: Supplementary file 1 — Figure S1. Representative VisCap visual outputs demonstrating (A) PKP2 exon 8 deletion, (B) RAF1 whole gene duplication, (C) Three copies of X chromosome. Table S1. Gene content of next‐generation sequencing panels. Table S2. Summary of patients included in the study. Table S3. Frequency of CNVs called by VisCap. [file MGG3-4-143-s001.docx]

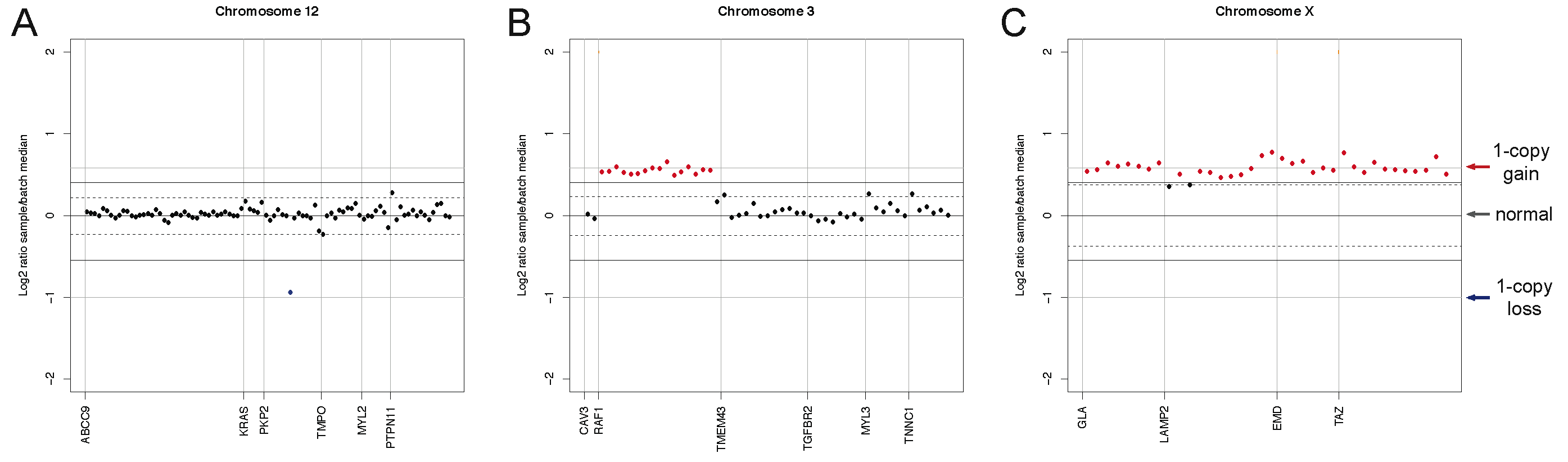


**Supplementary Figure S1:** Representative VisCap visual outputs demonstrating (A) PKP2 exon 8 deletion, (B) RAF1 whole gene duplication, (C) Three copies of X chromosome. Fraction of total coverage assigned to each interval within each sample were divided by the median for that target across the entire batch. log2 ratios were plotted by relative genome order. Each dot represents an exon. Expected log2 ratios for normal (copy number of 2), single-copy loss (leading to 1 copy of the target) and single-copy gain (leading to 3 copies of the target) are marked.

**Supplementary Table S1: Gene content of next-generation sequencing panels**

| **Test** | **Genes included in analysis** |
| --- | --- |
| PanCardiomyopathy Panel | ABCC9, ACTC1, ACTN2, ANKRD1, CASQ2, CAV3, CRYAB, CSRP3, CTF1, DES, DSC2, DSG2, DSP, DTNA, EMD, FHL2, GLA, JUP, LAMA4, LAMP2, LDB3, LMNA, MYBPC3, MYH6, MYH7, MYL2, MYL3, MYLK2, MYOZ2, NEXN, PKP2, PLN, PRKAG2, RBM20, RYR2, SGCD, TAZ, TCAP, TMEM43, TNNC1, TNNI3, TNNT2, TPM1, TTN, TTR, VCL |
| HCM Panel | ACTC1, ACTN2, CSRP3, GLA, LAMP2, MYBPC3, MYH7, MYL2, MYL3, MYOZ2, NEXN, PLN, PRKAG2, TNNC1, TNNI3, TNNT2, TPM1, TTR |
| DCM Panel | ABCC9, ACTC1, ACTN2, CSRP3, CTF1, DES, EMD, LAMP2, LDB3, LMNA, MYBPC3, MYH7, NEXN, PLN, RBM20, SGCD, TAZ, TCAP, TNNC1, TNNI3, TNNT2, TPM1, TTN, VCL |
| ARVC Panel | CASQ2, DSC2, DSG2, DSP, JUP, PKP2, RYR2, TMEM43 |
| LVNC Panel | ACTC1, CASQ2, DTNA, LDB3, LMNA, MYBPC3, MYH7, TAZ, TNNT2, VCL |

**Supplementary Table S2: Summary of patients included in the study**

| **Case ID** | **Clinical diagnosis** | **Panel tested** | **Sex** | **Age** | **Race/Ethnicity** | **Result prior to CNV analysis** |
| --- | --- | --- | --- | --- | --- | --- |
| Case1 | DCM | PanCardiomyopathy | Male | 37 yrs | White | Negative |
| Case2 | HCM | PanCardiomyopathy | Female | 60 yrs | White | Positive |
| Case3 | DCM | PanCardiomyopathy | Male | 55 yrs | White | Positive |
| Case4 | HCM | HCM | Male | 35 yrs | White | Positive |
| Case5 | HCM | HCM | Female | 41 yrs | Mixed | Negative |
| Case6 | HCM | HCM | Female | 50 yrs | Asian | Positive |
| Case7 | HCM | HCM | Male | 62 yrs | White | Negative |
| Case8 | HCM | HCM | Male | 60 yrs | White | Negative |
| Case9 | DCM | PanCardiomyopathy | Female | 77 yrs | White | Positive |
| Case10 | DCM | DCM | Male | 46 yrs | White | Positive |
| Case11 | HCM | PanCardiomyopathy | Male | 33 yrs | Black or African American | Inconclusive |
| Case12 | DCM | PanCardiomyopathy | Female | 28 yrs | White | Positive |
| Case13 | DCM | PanCardiomyopathy | Male | 65 yrs | White | Inconclusive |
| Case14 | ARVC | PanCardiomyopathy | Female | 62 yrs | White | Negative |
| Case15 | ARVC | PanCardiomyopathy | Female | 46 yrs | White | Inconclusive |
| Case16 | HCM | HCM | Female | 83 yrs | White | Negative |
| Case17 | HCM | PanCardiomyopathy | Male | 64 yrs | Unspecified | Inconclusive |
| Case18 | DCM | PanCardiomyopathy | Male | 54 yrs | White | Positive |
| Case19 | DCM, ARVC | PanCardiomyopathy | Female | 39 yrs | White | Inconclusive |
| Case20 | ARVC, HCM | PanCardiomyopathy | Male | 57 yrs | White | Negative |
| Case21 | HCM | HCM | Male | 51 yrs | White | Inconclusive |
| Case22 | ARVC, DCM | PanCardiomyopathy | Male | 29 yrs | Unspecified | Positive |
| Case23 | Cardiomyopathy NOS, Myopathy | PanCardiomyopathy | Female | 43 yrs | White | Positive |
| Case24 | DCM | PanCardiomyopathy | Male | 57 yrs | White | Positive |
| Case25 | HCM | PanCardiomyopathy | Female | 46 yrs | White | Positive |
| Case26 | DCM | PanCardiomyopathy | Female | 65 yrs | White | Inconclusive |
| Case27 | Cardiomyopathy NOS | PanCardiomyopathy | Male | 48 yrs | White | Positive |
| Case28 | HCM | HCM | Female | 71 yrs | Ashkenazi Jewish | Inconclusive |
| Case29 | HCM | HCM | Male | 50 yrs | White | Positive |
| Case30 | HCM | PanCardiomyopathy | Female | 30 yrs | White | Positive |
| Case31 | DCM | PanCardiomyopathy | Male | 69 yrs | Black or African American | Inconclusive |
| Case32 | DCM | PanCardiomyopathy | Female | 55 yrs | White | Negative |
| Case33 | DCM | PanCardiomyopathy | Male | 37 yrs | White | Positive |
| Case34 | LVNC | PanCardiomyopathy | Male | 58 yrs | White | Negative |
| Case35 | HCM | HCM | Male | 58 yrs | White | Positive |
| Case36 | HCM | PanCardiomyopathy | Female | 24 yrs | White | Inconclusive |
| Case37 | HCM | PanCardiomyopathy | Male | 48 yrs | White | Inconclusive |
| Case38 | DCM | PanCardiomyopathy | Male | 46 yrs | Asian | Inconclusive |
| Case39 | HCM | HCM | Female | 54 yrs | White | Negative |
| Case40 | DCM | PanCardiomyopathy | Female | 50 yrs | White | Positive |
| Case41 | DCM | PanCardiomyopathy | Female | 34 yrs | White | Positive |
| Case42 | HCM | PanCardiomyopathy | Male | 61 yrs | White | Inconclusive |
| Case43 | DCM | DCM | Female | 22 yrs | Unspecified | Positive |
| Case44 | HCM | HCM | Male | 34 yrs | Hispanic or Latino | Negative |
| Case45 | DCM | PanCardiomyopathy | Male | 55 yrs | White | Negative |
| Case46 | DCM | DCM | Male | 33 yrs | White | Positive |
| Case47 | ARVC | PanCardiomyopathy | Male | 53 yrs | White | Positive |
| Case48 | ARVC | PanCardiomyopathy | Male | 62 yrs | White | Inconclusive |
| Case49 | DCM | PanCardiomyopathy | Male | 60 yrs | White | Positive |
| Case50 | DCM | DCM | Male | 66 yrs | White | Positive |
| Case51 | DCM | PanCardiomyopathy | Male | 36 yrs | White | Inconclusive |
| Case52 | DCM | PanCardiomyopathy | Female | 65 yrs | White | Positive |
| Case53 | DCM | PanCardiomyopathy | Male | 62 yrs | White | Inconclusive |
| Case54 | HCM | PanCardiomyopathy | Female | 29 yrs | White | Negative |
| Case55 | HCM | PanCardiomyopathy | Male | 83 yrs | White | Inconclusive |
| Case56 | ARVC | ARVC | Female | 65 yrs | White | Negative |
| Case57 | ARVC | ARVC | Female | 65 yrs | White | Positive |
| Case58 | ARVC | ARVC | Male | 19 yrs | White | Positive |
| Case59 | ARVC | PanCardiomyopathy | Male | 24 yrs | White | Inconclusive |
| Case60 | HCM | HCM | Male | 63 yrs | Ashkenazi Jewish | Negative |
| Case61 | HCM | PanCardiomyopathy | Male | 57 yrs | Black or African American | Negative |
| Case62 | HCM | HCM | Male | 57 yrs | White | Negative |
| Case63 | ARVC | ARVC | Male | 54 yrs | White | Inconclusive |
| Case64 | DCM | PanCardiomyopathy | Male | 44 yrs | White | Positive |
| Case65 | DCM | PanCardiomyopathy | Male | 29 yrs | White | Inconclusive |
| Case66 | HCM | HCM | Male | 49 yrs | White | Negative |
| Case67 | ARVC | ARVC | Female | 54 yrs | White | Positive |
| Case68 | HCM | PanCardiomyopathy | Male | 57 yrs | White | Inconclusive |
| Case69 | HCM | HCM | Male | 29 yrs | Ashkenazi Jewish | Negative |
| Case70 | HCM | HCM | Male | 50 yrs | White | Positive |
| Case71 | DCM | PanCardiomyopathy | Male | 64 yrs | White | Inconclusive |
| Case72 | HCM | HCM | Female | 47 yrs | White | Positive |
| Case73 | DCM | PanCardiomyopathy | Male | 23 yrs | White | Inconclusive |
| Case74 | DCM | PanCardiomyopathy | Female | 21 yrs | White | Negative |
| Case75 | DCM | DCM | Male | 69 yrs | White | Negative |
| Case76 | ARVC | PanCardiomyopathy | Male | 61 yrs | White | Positive |
| Case77 | ARVC | PanCardiomyopathy | Male | 46 yrs | White | Positive |
| Case78 | HCM, LVNC | PanCardiomyopathy | Female | 21 yrs | White | Negative |
| Case79 | HCM | PanCardiomyopathy | Male | 25 yrs | Hispanic or Latino | Positive |
| Case80 | DCM | PanCardiomyopathy | Male | 29 yrs | Ashkenazi Jewish | Inconclusive |
| Case81 | DCM | DCM | Female | 49 yrs | White | Inconclusive |
| Case82 | DCM | PanCardiomyopathy | Male | 71 yrs | White | Inconclusive |
| Case83 | DCM | PanCardiomyopathy | Male | 63 yrs | Ashkenazi Jewish | Inconclusive |
| Case84 | HCM | PanCardiomyopathy | Female | 44 yrs | White | Positive |
| Case85 | DCM | PanCardiomyopathy | Male | 79 yrs | Unspecified | Inconclusive |
| Case86 | HCM | HCM | Female | 59 yrs | Black or African American | Negative |
| Case87 | DCM | PanCardiomyopathy | Female | 32 yrs | White | Inconclusive |
| Case88 | ARVC | ARVC | Female | 25 yrs | White | Negative |
| Case89 | ARVC | ARVC | Male | 69 yrs | White | Negative |
| Case90 | ARVC | ARVC | Male | 75 yrs | White | Negative |
| Case91 | HCM | HCM | Male | 43 yrs | White | Negative |
| Case92 | DCM | PanCardiomyopathy | Female | 34 yrs | Hispanic or Latino | Inconclusive |
| Case93 | DCM | DCM | Male | 41 yrs | White | Inconclusive |
| Case94 | DCM | PanCardiomyopathy | Female | 39 yrs | White | Inconclusive |
| Case95 | DCM | PanCardiomyopathy | Male | 64 yrs | White | Inconclusive |
| Case96 | DCM | DCM | Female | 66 yrs | White | Inconclusive |
| Case97 | ARVC | PanCardiomyopathy | Male | 17 yrs | Unspecified | Inconclusive |
| Case98 | DCM | PanCardiomyopathy | Female | 25 yrs | Unspecified | Positive |
| Case99 | DCM | PanCardiomyopathy | Male | 25 yrs | White | Inconclusive |
| Case100 | HCM | PanCardiomyopathy | Female | 73 yrs | White | Negative |
| Case101 | HCM | HCM | Female | 78 yrs | Ashkenazi Jewish | Negative |
| Case102 | HCM | HCM | Male | 68 yrs | White | Negative |
| Case103 | DCM | PanCardiomyopathy | Male | 48 yrs | White | Negative |
| Case104 | HCM | HCM | Male | 55 yrs | White | Inconclusive |
| Case105 | DCM | DCM | Male | 33 yrs | White | Positive |
| Case106 | DCM | PanCardiomyopathy | Male | 69 yrs | White | Inconclusive |
| Case107 | HCM | HCM | Male | 60 yrs | White | Negative |
| Case108 | Cardiomyopathy NOS | PanCardiomyopathy | Female | 61 yrs | White | Inconclusive |
| Case109 | HCM | HCM | Male | 29 yrs | White | Negative |
| Case110 | HCM | PanCardiomyopathy | Female | 51 yrs | White | Inconclusive |
| Case111 | HCM | HCM | Female | 69 yrs | White | Inconclusive |
| Case112 | DCM | PanCardiomyopathy | Male | 40 yrs | White | Positive |
| Case113 | HCM | PanCardiomyopathy | Male | 45 yrs | White | Inconclusive |
| Case114 | HCM | PanCardiomyopathy | Male | 10 yrs | White | Inconclusive |
| Case115 | HCM | HCM | Female | 75 yrs | White | Negative |
| Case116 | DCM | PanCardiomyopathy | Female | 56 yrs | White | Positive |
| Case117 | HCM | PanCardiomyopathy | Male | 4 yrs | White | Inconclusive |
| Case118 | HCM | HCM | Male | 46 yrs | White | Inconclusive |
| Case119 | DCM, Myopathy | PanCardiomyopathy | Male | 14 yrs | White | Inconclusive |
| Case120 | DCM | PanCardiomyopathy | Male | 4 yrs | White | Inconclusive |
| Case121 | DCM | PanCardiomyopathy | Male | 48 yrs | White | Positive |
| Case122 | DCM | PanCardiomyopathy | Male | 6 yrs | White | Inconclusive |
| Case123 | DCM | PanCardiomyopathy | Female | 26 yrs | White | Positive |
| Case124 | LVNC | DCM | Female | 4 yrs | Mixed | Negative |
| Case125 | DCM | PanCardiomyopathy | Female | 10 yrs | Asian | Inconclusive |
| Case126 | DCM | PanCardiomyopathy | Male | 4 yrs | White | Inconclusive |
| Case127 | DCM | PanCardiomyopathy | Male | 36 yrs | White | Inconclusive |
| Case128 | DCM | PanCardiomyopathy | Male | 45 yrs | White | Inconclusive |
| Case129 | Cardiomyopathy NOS | PanCardiomyopathy | Male | 11 yrs | White | Inconclusive |
| Case130 | DCM | PanCardiomyopathy | Female | 4 yrs | White | Inconclusive |
| Case131 | DCM | PanCardiomyopathy | Unspecified | 66 yrs | White | Negative |
| Case132 | DCM | PanCardiomyopathy | Male | 42 yrs | White | Negative |
| Case133 | DCM | PanCardiomyopathy | Male | 3 yrs | White | Negative |
| Case134 | RCM | PanCardiomyopathy | Male | 17 yrs | White | Inconclusive |
| Case135 | ARVC | PanCardiomyopathy | Female | 29 yrs | Unspecified | Inconclusive |
| Case136 | DCM | DCM | Male | 61 yrs | Ashkenazi Jewish | Positive |
| Case137 | HCM | HCM | Male | 16 yrs | White | Inconclusive |
| Case138 | HCM | PanCardiomyopathy | Female | 27 yrs | White | Inconclusive |
| Case139 | DCM | PanCardiomyopathy | Male | 21 yrs | Unspecified | Inconclusive |
| Case140 | HCM | PanCardiomyopathy | Female | 1 yrs | White | Inconclusive |
| Case141 | DCM | PanCardiomyopathy | Male | 45 yrs | Asian | Positive |
| Case142 | HCM | PanCardiomyopathy | Male | 2 yrs | White | Negative |
| Case143 | ARVC | PanCardiomyopathy | Female | 58 yrs | White | Inconclusive |
| Case144 | HCM | PanCardiomyopathy | Female | 2 yrs | White | Inconclusive |
| Case145 | DCM | PanCardiomyopathy | Male | 70 yrs | White | Inconclusive |
| Case146 | DCM | PanCardiomyopathy | Male | 23 yrs | Asian | Inconclusive |
| Case147 | HCM | HCM | Female | 45 yrs | Asian | Positive |
| Case148 | HCM | HCM | Male | 68 yrs | White | Negative |
| Case149 | HCM | PanCardiomyopathy | Male | 5 yrs | Asian | Inconclusive |
| Case150 | DCM | DCM | Female | 1 mos | Mixed | Inconclusive |
| Case151 | HCM | HCM | Female | 71 yrs | Ashkenazi Jewish | Negative |
| Case152 | LVNC | PanCardiomyopathy | Female | 29 yrs | Unspecified | Inconclusive |
| Case153 | HCM | HCM | Female | 1 mos | White | Inconclusive |
| Case154 | HCM | HCM | Unknown | 33 yrs | Unspecified | Negative |
| Case155 | HCM | PanCardiomyopathy | Female | 61 yrs | Asian | Inconclusive |
| Case156 | HCM | HCM | Male | 3 mos | White | Inconclusive |
| Case157 | HCM | PanCardiomyopathy | Male | 62 yrs | White | Inconclusive |
| Case158 | HCM | PanCardiomyopathy | Male | 55 yrs | Ashkenazi Jewish | Inconclusive |
| Case159 | LVNC, Myopathy | PanCardiomyopathy | Male | 15 yrs | Unspecified | Negative |
| Case160 | LVNC | PanCardiomyopathy | Male | 39 yrs | White | Positive |
| Case161 | HCM | HCM | Unknown | 211 yrs | White | Positive |
| Case162 | HCM | PanCardiomyopathy | Male | 12 yrs | Unspecified | Inconclusive |
| Case163 | HCM | HCM | Male | 38 yrs | White | Positive |
| Case164 | HCM | HCM | Male | 28 yrs | Unspecified | Negative |
| Case165 | HCM | HCM | Male | 48 yrs | Asian | Negative |
| Case166 | DCM | PanCardiomyopathy | Female | 1 yrs | Black or African American | Negative |
| Case167 | HCM | PanCardiomyopathy | Female | 27 yrs | White | Positive |
| Case168 | DCM | PanCardiomyopathy | Female | 59 yrs | White | Positive |
| Case169 | HCM | HCM | Male | 47 yrs | White | Positive |
| Case170 | ARVC | PanCardiomyopathy | Male | 15 yrs | White | Inconclusive |
| Case171 | HCM | HCM | Male | 46 yrs | Black or African American | Negative |
| Case172 | HCM | HCM | Male | 64 yrs | Black or African American | Positive |
| Case173 | DCM | PanCardiomyopathy | Male | 10 yrs | Asian | Positive |
| Case174 | DCM | PanCardiomyopathy | Female | 55 yrs | White | Positive |
| Case175 | HCM | HCM | Female | 52 yrs | White | Negative |
| Case176 | DCM | DCM | Male | 17 yrs | Black or African American | Inconclusive |
| Case177 | HCM | HCM | Male | 50 yrs | White | Inconclusive |
| Case178 | ARVC | PanCardiomyopathy | Male | 22 yrs | White | Positive |
| Case179 | DCM | PanCardiomyopathy | Female |  | Hispanic or Latino | Inconclusive |
| Case180 | HCM | HCM | Male | 69 yrs | Unspecified | Inconclusive |
| Case181 | HCM | HCM | Female | 77 yrs | Black or African American | Positive |
| Case182 | HCM | HCM | Female | 74 yrs | White | Negative |
| Case183 | HCM | PanCardiomyopathy | Female | 82 yrs | Asian | Inconclusive |
| Case184 | LVNC | PanCardiomyopathy | Male | 3 yrs | Unspecified | Inconclusive |
| Case185 | HCM | HCM | Male | 36 yrs | White | Negative |
| Case186 | ARVC | ARVC | Male | 15 yrs | Unspecified | Inconclusive |
| Case187 | HCM | HCM | Male | 45 yrs | White | Positive |
| Case188 | HCM | HCM | Female | 75 yrs | White | Negative |
| Case189 | DCM | PanCardiomyopathy | Male | 1 yrs | White | Positive |
| Case190 | DCM | PanCardiomyopathy | Male | 15 yrs | Hispanic or Latino | Inconclusive |
| Case191 | HCM | PanCardiomyopathy | Male | 22 yrs | Black or African American | Inconclusive |
| Case192 | DCM | PanCardiomyopathy | Female | 46 yrs | White | Inconclusive |
| Case193 | LVNC | LVNC | Male | 51 yrs | White | Negative |
| Case194 | HCM | PanCardiomyopathy | Female | 77 yrs | Unspecified | Negative |
| Case195 | HCM | PanCardiomyopathy | Male | 57 yrs | Unspecified | Negative |
| Case196 | HCM | PanCardiomyopathy | Female | 80 yrs | Unspecified | Negative |
| Case197 | HCM | PanCardiomyopathy | Female | 37 yrs | Unspecified | Negative |
| Case198 | HCM | PanCardiomyopathy | Male | 73 yrs | Unspecified | Negative |
| Case199 | HCM | PanCardiomyopathy | Female | 32 yrs | Unspecified | Negative |
| Case200 | HCM | PanCardiomyopathy | Female | 59 yrs | Unspecified | Negative |
| Case201 | HCM | PanCardiomyopathy | Male | 70 yrs | Unspecified | Negative |
| Case202 | HCM | PanCardiomyopathy | Male | 66 yrs | Unspecified | Negative |
| Case203 | HCM | PanCardiomyopathy | Unknown | 13 yrs | Black or African American | Positive |
| Case204 | HCM | HCM | Male | 69 yrs | Unspecified | Negative |
| Case205 | HCM | PanCardiomyopathy | Male | 46 yrs | White | Inconclusive |
| Case206 | HCM | HCM | Female | 77 yrs | Unspecified | Positive |
| Case207 | HCM | HCM | Male | 9 yrs | Hispanic or Latino | Negative |
| Case208 | DCM | PanCardiomyopathy | Female | 1 yrs | Black or African American | Inconclusive |
| Case209 | DCM | PanCardiomyopathy | Female | 4 mos | White | Negative |
| Case210 | HCM | HCM | Female | 74 yrs | White | Inconclusive |
| Case211 | HCM, LVNC | PanCardiomyopathy | Male | 77 yrs | Black or African American | Inconclusive |
| Case212 | HCM | HCM | Male | 54 yrs | White | Negative |
| Case213 | ARVC | ARVC | Male | 48 yrs | Unspecified | Positive |
| Case214 | DCM | PanCardiomyopathy | Female | 24 yrs | White | Positive |
| Case215 | DCM | PanCardiomyopathy | Female |  | Black or African American | Inconclusive |
| Case216 | HCM | PanCardiomyopathy | Male | 51 yrs | White | Positive |
| Case217 | RCM | PanCardiomyopathy | Female | 10 yrs | Unspecified | Inconclusive |
| Case218 | HCM | PanCardiomyopathy | Male | 39 yrs | White | Positive |
| Case219 | DCM | DCM | Male | 53 yrs | White | Negative |
| Case220 | HCM | HCM | Female | 63 yrs | Unspecified | Negative |
| Case221 | HCM | HCM | Male | 77 yrs | White | Negative |
| Case222 | ARVC | PanCardiomyopathy | Male | 17 yrs | Black or African American | Inconclusive |
| Case223 | HCM | HCM | Male | 73 yrs | Black or African American | Negative |
| Case224 | HCM | PanCardiomyopathy | Male | 30 yrs | Mixed | Inconclusive |
| Case225 | HCM | HCM | Male | 68 yrs | White | Positive |
| Case226 | HCM | HCM | Unknown | 55 yrs | White | Negative |
| Case227 | DCM | PanCardiomyopathy | Male | 42 yrs | White | Negative |
| Case228 | DCM | DCM | Female | 1 yrs | Black or African American | Inconclusive |
| Case229 | DCM | DCM | Female | 1 mos | White | Inconclusive |
| Case230 | HCM, Myopathy | PanCardiomyopathy | Female | 7 mos | Unspecified | Positive |
| Case231 | HCM | PanCardiomyopathy | Male | 21 yrs | Black or African American | Inconclusive |
| Case232 | HCM | HCM | Male | 1 yrs | Black or African American | Inconclusive |
| Case233 | HCM | HCM | Female | 69 yrs | White | Inconclusive |
| Case234 | HCM | HCM | Male | 58 yrs | White | Inconclusive |
| Case235 | HCM | HCM | Male | 62 yrs | White | Positive |
| Case236 | HCM | PanCardiomyopathy | Male | 47 yrs | Unspecified | Inconclusive |
| Case237 | DCM | PanCardiomyopathy | Male | 65 yrs | White | Negative |
| Case238 | HCM | PanCardiomyopathy | Unknown | 60 yrs | White | Negative |
| Case239 | HCM | HCM | Unknown | 51 yrs | White | Negative |
| Case240 | HCM | HCM | Female | 73 yrs | White | Positive |
| Case241 | HCM | HCM | Male | 39 yrs | White | Positive |
| Case242 | LVNC | LVNC | Male | 39 yrs | Mixed | Negative |
| Case243 | DCM | PanCardiomyopathy | Female | 6 yrs | White | Inconclusive |
| Case244 | DCM | PanCardiomyopathy | Female | 30 yrs | White | Positive |
| Case245 | HCM | HCM | Unknown | 77 yrs | White | Negative |
| Case246 | DCM | PanCardiomyopathy | Female | 35 yrs | Unspecified | Inconclusive |
| Case247 | RCM | PanCardiomyopathy | Male | 16 yrs | White | Inconclusive |
| Case248 | DCM | PanCardiomyopathy | Male | 16 yrs | Asian | Inconclusive |
| Case249 | HCM | PanCardiomyopathy | Female | 44 yrs | White | Positive |
| Case250 | DCM | PanCardiomyopathy | Male | 21 yrs | Unspecified | Inconclusive |
| Case251 | HCM | HCM | Unknown | 46 yrs | Unspecified | Negative |
| Case252 | DCM | DCM | Male | 2 mos | Unspecified | Negative |
| Case253 | HCM | PanCardiomyopathy | Male | 4 mos | Black or African American | Inconclusive |
| Case254 | HCM | HCM | Female | 2 mos | White | Negative |
| Case255 | HCM | PanCardiomyopathy | Female | 72 yrs | White | Inconclusive |
| Case256 | HCM | HCM | Male | 58 yrs | Unspecified | Positive |
| Case257 | ARVC | ARVC | Male | 47 yrs | White | Negative |
| Case258 | HCM | PanCardiomyopathy | Male | 8 yrs | Black or African American | Inconclusive |
| Case259 | DCM | PanCardiomyopathy | Female | 9 yrs | Unspecified | Positive |
| Case260 | DCM | PanCardiomyopathy | Female | 2 yrs | Asian | Inconclusive |
| Case261 | DCM | PanCardiomyopathy | Male | 6 mos | White | Inconclusive |
| Case262 | HCM | HCM | Male | 21 yrs | Unspecified | Positive |
| Case263 | LVNC | PanCardiomyopathy | Unknown | 17 yrs | Black or African American | Negative |
| Case264 | HCM | HCM | Female | 16 yrs | White | Positive |
| Case265 | ARVC | ARVC | Male | 56 yrs | White | Inconclusive |
| Case266 | DCM | PanCardiomyopathy | Female | 4 yrs | Mixed | Positive |
| Case267 | HCM | HCM | Female | 44 yrs | White | Negative |
| Case268 | HCM | HCM | Male | 60 yrs | White | Negative |
| Case269 | HCM | HCM | Male | 46 yrs | Unspecified | Positive |
| Case270 | HCM | HCM | Female | 58 yrs | Unspecified | Inconclusive |
| Case271 | HCM | HCM | Female | 47 yrs | White | Positive |
| Case272 | HCM | HCM | Male | 1 mos | White | Inconclusive |
| Case273 | HCM | HCM | Male | 49 yrs | Unspecified | Negative |
| Case274 | Cardiomyopathy NOS | PanCardiomyopathy | Male | 34 yrs | Black or African American | Inconclusive |
| Case275 | HCM | HCM | Female | 67 yrs | White | Positive |
| Case276 | HCM | HCM | Female | 61 yrs | White | Inconclusive |
| Case277 | HCM | HCM | Male | 36 yrs | White | Positive |
| Case278 | ARVC | PanCardiomyopathy | Male | 42 yrs | White | Inconclusive |
| Case279 | HCM | PanCardiomyopathy | Male | 48 yrs | White | Negative |
| Case280 | HCM | HCM | Female | 29 yrs | White | Positive |
| Case281 | HCM | HCM | Male | 16 yrs | White | Positive |
| Case282 | HCM | PanCardiomyopathy | Male | 41 yrs | White | Positive |
| Case283 | HCM | HCM | Male | 46 yrs | White | Inconclusive |
| Case284 | LVNC | PanCardiomyopathy | Female | 8 mos | Unspecified | Inconclusive |
| Case285 | HCM | HCM | Female | 20 yrs | Black or African American | Negative |
| Case286 | DCM | DCM | Unknown | 75 yrs | Unspecified | Negative |
| Case287 | DCM | DCM | Female | 63 yrs | White | Inconclusive |
| Case288 | DCM | DCM | Male | 62 yrs | Unspecified | Negative |
| Case289 | HCM | PanCardiomyopathy | Male | 67 yrs | Black or African American | Positive |
| Case290 | ARVC | PanCardiomyopathy | Female | 46 yrs | White | Negative |
| Case291 | HCM | PanCardiomyopathy | Male | 35 yrs | Ashkenazi Jewish | Positive |
| Case292 | HCM | HCM | Female | 56 yrs | Asian | Inconclusive |
| Case293 | HCM | HCM | Male | 24 yrs | Black or African American | Negative |
| Case294 | DCM | PanCardiomyopathy | Female | 8 mos | Asian | Inconclusive |
| Case295 | DCM | PanCardiomyopathy | Male | 15 yrs | White | Inconclusive |
| Case296 | DCM | PanCardiomyopathy | Female | 57 yrs | White | Positive |
| Case297 | HCM | PanCardiomyopathy | Male | 54 yrs | White | Inconclusive |
| Case298 | HCM | HCM | Female | 16 yrs | White | Inconclusive |
| Case299 | HCM | PanCardiomyopathy | Female | 27 yrs | White | Inconclusive |
| Case300 | DCM | PanCardiomyopathy | Male | 24 yrs | Black or African American | Inconclusive |
| Case301 | DCM | PanCardiomyopathy | Male | 43 yrs | White | Negative |
| Case302 | DCM, LVNC | PanCardiomyopathy | Male | 1 mos | Unspecified | Inconclusive |
| Case303 | DCM | PanCardiomyopathy | Unknown | 49 yrs | Black or African American | Inconclusive |
| Case304 | DCM | DCM | Male | 1 yrs | White | Inconclusive |
| Case305 | DCM | PanCardiomyopathy | Male | 24 yrs | Unspecified | Inconclusive |
| Case306 | HCM | PanCardiomyopathy | Female | 69 yrs | White | Inconclusive |
| Case307 | DCM | DCM | Female | 3 mos | Black or African American | Inconclusive |
| Case308 | Cardiomyopathy NOS | PanCardiomyopathy | Male | 2 mos | White | Inconclusive |
| Case309 | ARVC | ARVC | Male | 56 yrs | White | Positive |
| Case310 | HCM | HCM | Male | 68 yrs | White | Positive |
| Case311 | HCM | HCM | Female | 59 yrs | White | Negative |
| Case312 | HCM | HCM | Unknown | 42 yrs | Unspecified | Negative |
| Case313 | HCM | HCM | Female | 3 mos | Black or African American |  |
| Case314 | HCM | PanCardiomyopathy | Male |  | White | Inconclusive |
| Case315 | HCM | HCM | Male | 53 yrs | White | Negative |
| Case316 | DCM | PanCardiomyopathy | Male | 14 yrs | White | Inconclusive |
| Case317 | HCM | HCM | Male | 57 yrs | White | Negative |
| Case318 | DCM | DCM | Unknown | 1 mos | Unspecified | Inconclusive |
| Case319 | DCM | DCM | Female | 31 yrs | White | Inconclusive |
| Case320 | HCM | HCM | Male | 36 yrs | Unspecified | Positive |
| Case321 | DCM | PanCardiomyopathy | Female | 33 yrs | White | Inconclusive |
| Case322 | LVNC | PanCardiomyopathy | Male | 1 yrs | Black or African American | Inconclusive |
| Case323 | HCM | HCM | Male | 19 yrs | White | Inconclusive |
| Case324 | DCM | PanCardiomyopathy | Female | 68 yrs | Unspecified | Positive |
| Case325 | DCM | PanCardiomyopathy | Female | 11 yrs | Unspecified | Inconclusive |
| Case326 | DCM | PanCardiomyopathy | Female | 10 mos | Black or African American | Inconclusive |
| Case327 | HCM | HCM | Unknown | 56 yrs | Unspecified | Negative |
| Case328 | HCM | PanCardiomyopathy | Male | 73 yrs | White | Negative |
| Case329 | DCM | PanCardiomyopathy | Male | 2 yrs | Unspecified | Inconclusive |
| Case330 | ARVC | ARVC | Female | 53 yrs | White | Positive |
| Case331 | HCM | HCM | Male | 37 yrs | White | Positive |
| Case332 | HCM | HCM | Male | 46 yrs | Ashkenazi Jewish | Negative |
| Case333 | HCM | PanCardiomyopathy | Female | 45 yrs | Unspecified | Inconclusive |
| Case334 | HCM | PanCardiomyopathy | Male | 43 yrs | Unspecified | Inconclusive |
| Case335 | DCM | PanCardiomyopathy | Male | 16 yrs | White | Inconclusive |
| Case336 | DCM | PanCardiomyopathy | Male | 14 yrs | White | Inconclusive |
| Case337 | HCM | HCM | Male | 7 mos | Black or African American | Inconclusive |
| Case338 | HCM | HCM | Male | 16 yrs | Hispanic or Latino | Negative |
| Case339 | HCM | HCM | Male | 40 yrs | White | Negative |
| Case340 | DCM | PanCardiomyopathy | Male | 41 yrs | White | Inconclusive |
| Case341 | DCM | PanCardiomyopathy | Female | 33 yrs | White | Inconclusive |
| Case342 | DCM | PanCardiomyopathy | Male | 2 mos | White | Inconclusive |
| Case343 | HCM | HCM | Male | 44 yrs | White | Positive |
| Case344 | DCM | DCM | Male | 43 yrs | White | Inconclusive |
| Case345 | DCM | PanCardiomyopathy | Female | 35 yrs | White | Inconclusive |
| Case346 | LVNC | PanCardiomyopathy | Male | 1 yrs | White | Positive |
| Case347 | ARVC | ARVC | Male | 9 yrs | Unspecified | Negative |
| Case348 | LVNC | PanCardiomyopathy | Female | 42 yrs | Unspecified | Inconclusive |
| Case349 | DCM | PanCardiomyopathy | Male | 4 mos | Black or African American | Inconclusive |
| Case350 | HCM | HCM | Male | 21 yrs | White | Inconclusive |
| Case351 | HCM | HCM | Female | 72 yrs | White | Positive |
| Case352 | HCM | PanCardiomyopathy | Male | 46 yrs | White | Positive |
| Case353 | HCM | HCM | Male | 65 yrs | White | Negative |
| Case354 | LVNC | PanCardiomyopathy | Female | 5 yrs | White | Inconclusive |
| Case355 | DCM | DCM | Unknown | 58 yrs | White | Positive |
| Case356 | HCM | PanCardiomyopathy | Female | 47 yrs | Unspecified | Positive |
| Case357 | HCM | HCM | Male | 74 yrs | White | Negative |
| Case358 | HCM | HCM | Male | 76 yrs | White | Negative |
| Case359 | HCM | HCM | Male | 30 yrs | White | Negative |
| Case360 | ARVC | ARVC | Female | 14 yrs | Asian | Inconclusive |
| Case361 | HCM | HCM | Male | 16 yrs | Unspecified | Inconclusive |
| Case362 | DCM | DCM | Male | 1 yrs | White | Negative |
| Case363 | DCM | PanCardiomyopathy | Female | 35 yrs | White | Inconclusive |
| Case364 | DCM | PanCardiomyopathy | Unknown | 38 yrs | Unspecified | Positive |
| Case365 | HCM | PanCardiomyopathy | Male | 38 yrs | Unspecified | Positive |
| Case366 | HCM | HCM | Female | 51 yrs | White | Positive |
| Case367 | HCM | PanCardiomyopathy | Male | 31 yrs | Native Hawaiian or Other Pacific Islander | Positive |
| Case368 | HCM | PanCardiomyopathy | Male | 39 yrs | White | Positive |
| Case369 | ARVC | ARVC | Male | 52 yrs | White | Inconclusive |
| Case370 | Cardiomyopathy NOS | PanCardiomyopathy | Unknown | 1 yrs | Black or African American | Inconclusive |
| Case371 | HCM | HCM | Male | 58 yrs | White | Negative |
| Case372 | HCM | HCM | Male | 37 yrs | White | Positive |
| Case373 | HCM | HCM | Female | 81 yrs | White | Negative |
| Case374 | DCM, (Myopathy) | PanCardiomyopathy | Male | 26 yrs | Unspecified | Positive |
| Case375 | RCM | HCM | Male | 5 yrs | Unspecified | Negative |
| Case376 | HCM | HCM | Female | 59 yrs | Unspecified | Inconclusive |
| Case377 | Cardiomyopathy NOS | PanCardiomyopathy | Male | 55 yrs | Black or African American | Inconclusive |
| Case378 | DCM | PanCardiomyopathy | Female | 41 yrs | White | Positive |
| Case379 | HCM | PanCardiomyopathy | Female | 8 mos | White | Inconclusive |
| Case380 | ARVC | ARVC | Female | 58 yrs | White | Negative |
| Case381 | DCM | PanCardiomyopathy | Female | 60 yrs | White | Positive |
| Case382 | DCM, LVNC | PanCardiomyopathy | Male | 22 yrs | White | Positive |
| Case383 | DCM | DCM | Female | 7 mos | White | Inconclusive |
| Case384 | HCM | HCM | Female | 42 yrs | Black or African American | Positive |
| Case385 | HCM | HCM | Male | 65 yrs | Unspecified | Negative |
| Case386 | Cardiomyopathy NOS | PanCardiomyopathy | Male | 51 yrs | White | Inconclusive |
| Case387 | HCM | PanCardiomyopathy | Male | 38 yrs | Unspecified | Inconclusive |
| Case388 | ARVC | PanCardiomyopathy | Female | 68 yrs | White | Inconclusive |
| Case389 | HCM | HCM | Male | 65 yrs | White | Negative |
| Case390 | HCM | HCM | Male | 55 yrs | White | Negative |
| Case391 | HCM | HCM | Female | 68 yrs | White | Inconclusive |
| Case392 | HCM | HCM | Female | 53 yrs | White | Positive |
| Case393 | HCM | HCM | Female | 48 yrs | White | Positive |
| Case394 | HCM | PanCardiomyopathy | Female | 65 yrs | White | Inconclusive |
| Case395 | HCM | HCM | Male | 1 mos | White | Positive |
| Case396 | HCM | HCM | Male | 69 yrs | White | Inconclusive |
| Case397 | HCM | HCM | Female | 68 yrs | White | Inconclusive |
| Case398 | DCM | PanCardiomyopathy | Female | 35 yrs | Unspecified | Inconclusive |
| Case399 | DCM | PanCardiomyopathy | Male | 33 yrs | White | Inconclusive |
| Case400 | DCM | PanCardiomyopathy | Female | 52 yrs | White | Inconclusive |
| Case401 | DCM | PanCardiomyopathy | Male | 23 yrs | White | Inconclusive |
| Case402 | HCM | HCM | Male | 42 yrs | Unspecified | Positive |
| Case403 | LVNC | LVNC | Female | 43 yrs | White | Negative |
| Case404 | ARVC | ARVC | Male | 57 yrs | White | Negative |
| Case405 | HCM | PanCardiomyopathy | Female | 31 yrs | Unspecified | Inconclusive |
| Case406 | HCM | PanCardiomyopathy | Male | 58 yrs | White | Negative |
| Case407 | DCM | PanCardiomyopathy | Female | 51 yrs | White | Inconclusive |
| Case408 | DCM | PanCardiomyopathy | Male | 41 yrs | White | Positive |
| Case409 | DCM, LVNC | PanCardiomyopathy | Male | 27 yrs | Mixed | Positive |
| Case410 | DCM | PanCardiomyopathy | Female | 66 yrs | Black or African American | Negative |
| Case411 | DCM, LVNC | PanCardiomyopathy | Female | 9 yrs | Mixed | Positive |
| Case412 | HCM | HCM | Female | 35 yrs | White | Positive |
| Case413 | HCM | PanCardiomyopathy | Male | 62 yrs | Black or African American | Inconclusive |
| Case414 | DCM | PanCardiomyopathy | Male | 49 yrs | White | Positive |
| Case415 | HCM | HCM | Male | 67 yrs | White | Negative |
| Case416 | ARVC | ARVC | Female | 52 yrs | White | Negative |
| Case417 | HCM | PanCardiomyopathy | Male | 4 mos | White | Inconclusive |
| Case418 | HCM | PanCardiomyopathy | Female | 56 yrs | White | Positive |
| Case419 | RCM | PanCardiomyopathy | Male | 8 yrs | White | Positive |
| Case420 | HCM | PanCardiomyopathy | Female | 61 yrs | Ashkenazi Jewish | Inconclusive |
| Case421 | HCM | PanCardiomyopathy | Male | 41 yrs | Ashkenazi Jewish | Inconclusive |
| Case422 | LVNC | LVNC | Male | 15 yrs | Unspecified | Negative |
| Case423 | HCM | HCM | Female | 11 yrs | Black or African American | Negative |
| Case424 | DCM | PanCardiomyopathy | Female | 7 mos | White | Inconclusive |
| Case425 | DCM | PanCardiomyopathy | Male | 20 yrs | White | Inconclusive |
| Case426 | HCM | HCM | Female |  | White | Negative |
| Case427 | Cardiomyopathy NOS | PanCardiomyopathy | Male | 58 yrs | White | Inconclusive |
| Case428 | DCM | PanCardiomyopathy | Female | 67 yrs | Unspecified | Inconclusive |
| Case429 | RCM | PanCardiomyopathy | Male | 17 yrs | White | Positive |
| Case430 | ARVC | ARVC | Female | 53 yrs | White | Inconclusive |
| Case431 | HCM | PanCardiomyopathy | Unknown | 2 mos | Hispanic or Latino | Inconclusive |
| Case432 | HCM | HCM | Male | 48 yrs | White | Positive |
| Case433 | DCM | PanCardiomyopathy | Female | 3 yrs | White | Inconclusive |
| Case434 | HCM | HCM | Male | 15 yrs | Unspecified | Positive |
| Case435 | HCM | PanCardiomyopathy | Female | 35 yrs | White | Positive |
| Case436 | LVNC | LVNC | Male |  | White | Negative |
| Case437 | DCM | PanCardiomyopathy | Female | 10 mos | Asian | Inconclusive |
| Case438 | DCM | PanCardiomyopathy | Female | 32 yrs | White | Inconclusive |
| Case439 | DCM | DCM | Female | 6 mos | Hispanic or Latino | Inconclusive |
| Case440 | Cardiomyopathy NOS | PanCardiomyopathy | Female | 50 yrs | White | Inconclusive |
| Case441 | DCM | PanCardiomyopathy | Unknown | 26 yrs | White | Inconclusive |
| Case442 | HCM | HCM | Male | 46 yrs | Black or African American | Positive |
| Case443 | DCM | DCM | Male | 23 yrs | White | Positive |
| Case444 | HCM | HCM | Female | 47 yrs | Unspecified | Inconclusive |
| Case445 | HCM | HCM | Female | 36 yrs | Unspecified | Negative |
| Case446 | LVNC | PanCardiomyopathy | Male | 31 yrs | Black or African American | Positive |
| Case447 | RCM | PanCardiomyopathy | Male | 10 mos | Unspecified | Inconclusive |
| Case448 | HCM | HCM | Male | 69 yrs | White | Negative |
| Case449 | DCM | PanCardiomyopathy | Male | 5 yrs | White | Positive |
| Case450 | DCM | DCM | Male | 49 yrs | White | Inconclusive |
| Case451 | DCM | PanCardiomyopathy | Female | 1 yrs | White | Inconclusive |
| Case452 | DCM | PanCardiomyopathy | Male | 11 yrs | White | Positive |
| Case453 | HCM | HCM | Male | 77 yrs | White | Negative |
| Case454 | HCM | HCM | Male | 47 yrs | Unspecified | Positive |
| Case455 | HCM | HCM | Female | 80 yrs | White | Inconclusive |
| Case456 | HCM | PanCardiomyopathy | Female | 2 mos | Black or African American | Positive |
| Case457 | HCM | HCM | Male | 63 yrs | White | Negative |
| Case458 | DCM | DCM | Female | 17 yrs | White | Positive |
| Case459 | DCM | DCM | Female | 4 mos | White | Inconclusive |
| Case460 | Cardiomyopathy NOS | PanCardiomyopathy | Male | 9 mos | Unspecified | Inconclusive |
| Case461 | HCM | PanCardiomyopathy | Female | 4 yrs | Unspecified | Inconclusive |
| Case462 | HCM | HCM | Male | 4 yrs | Mixed | Positive |
| Case463 | DCM | PanCardiomyopathy | Male | 9 yrs | Black or African American | Inconclusive |
| Case464 | HCM | HCM | Female | 28 yrs | Unspecified | Negative |
| Case465 | HCM | PanCardiomyopathy | Male | 54 yrs | Unspecified | Negative |
| Case466 | DCM | PanCardiomyopathy | Male | 6 yrs | Black or African American | Inconclusive |
| Case467 | HCM | PanCardiomyopathy | Male | 79 yrs | Ashkenazi Jewish | Inconclusive |
| Case468 | HCM | PanCardiomyopathy | Male | 5 yrs | Unspecified | Inconclusive |
| Case469 | Cardiomyopathy NOS | PanCardiomyopathy | Male | 17 yrs | White | Inconclusive |
| Case470 | DCM | PanCardiomyopathy | Female | 7 mos | Black or African American | Inconclusive |
| Case471 | HCM | PanCardiomyopathy | Male | 17 yrs | White | Inconclusive |
| Case472 | DCM | PanCardiomyopathy | Female | 67 yrs | White | Inconclusive |
| Case473 | HCM | PanCardiomyopathy | Female | 58 yrs | White | Positive |
| Case474 | LVNC | PanCardiomyopathy | Male | 1 mos | Black or African American | Positive |
| Case475 | HCM | HCM | Male | 45 yrs | Ashkenazi Jewish | Positive |
| Case476 | HCM | HCM | Male | 26 yrs | Mixed | Inconclusive |
| Case477 | ARVC | PanCardiomyopathy | Female | 31 yrs | White | Inconclusive |
| Case478 | DCM | PanCardiomyopathy | Male | 1 mos | White | Inconclusive |
| Case479 | DCM, LVNC | PanCardiomyopathy | Male | 2 mos | White | Positive |
| Case480 | HCM | PanCardiomyopathy | Male | 35 yrs | White | Negative |
| Case481 | DCM | HCM | Female | 35 yrs | Unspecified | Positive |
| Case482 | LVNC | PanCardiomyopathy | Female | 1 mos | White | Inconclusive |
| Case483 | DCM | DCM | Male | 2 yrs | Unspecified | Positive |
| Case484 | HCM | HCM | Male | 28 yrs | White | Positive |
| Case485 | Cardiomyopathy NOS | PanCardiomyopathy | Female | 61 yrs | Ashkenazi Jewish | Inconclusive |
| Case486 | HCM | HCM | Female | 6 mos | Unspecified | Inconclusive |
| Case487 | LVNC | LVNC | Female | 28 yrs | Ashkenazi Jewish | Inconclusive |
| Case488 | DCM | PanCardiomyopathy | Female | 61 yrs | Mixed | Positive |
| Case489 | DCM | PanCardiomyopathy | Male | 16 yrs | Asian | Inconclusive |
| Case490 | DCM | PanCardiomyopathy | Male | 23 yrs | Unspecified | Inconclusive |
| Case491 | HCM | HCM | Male | 15 yrs | Hispanic or Latino | Negative |
| Case492 | HCM | PanCardiomyopathy | Female |  | Hispanic or Latino | Inconclusive |
| Case493 | DCM | PanCardiomyopathy | Female | 15 yrs | White | Inconclusive |
| Case494 | DCM | PanCardiomyopathy | Male | 10 yrs | Hispanic or Latino | Inconclusive |
| Case495 | DCM | PanCardiomyopathy | Male | 8 mos | White | Inconclusive |
| Case496 | HCM | PanCardiomyopathy | Male | 73 yrs | Ashkenazi Jewish | Inconclusive |
| Case497 | LVNC | LVNC | Male | 1 yrs | White | Negative |
| Case498 | HCM | HCM | Male | 65 yrs | White | Negative |
| Case499 | HCM | HCM | Female | 12 yrs | Black or African American | Negative |
| Case500 | DCM | PanCardiomyopathy | Male | 7 mos | Unspecified | Inconclusive |
| Case501 | HCM | HCM | Female | 39 yrs | Unspecified | Positive |
| Case502 | HCM | PanCardiomyopathy | Male | 40 yrs | Native Hawaiian or Other Pacific Islander | Inconclusive |
| Case503 | HCM | HCM | Male | 30 yrs | Black or African American | Negative |
| Case504 | DCM, LVNC | DCM | Male | 32 yrs | Black or African American | Positive |
| Case505 | RCM | PanCardiomyopathy | Male | 18 yrs | White | Positive |
| Case506 | LVNC | PanCardiomyopathy | Male | 15 yrs | Black or African American | Inconclusive |
| Case507 | HCM | PanCardiomyopathy | Male | 45 yrs | White | Positive |
| Case508 | LVNC | PanCardiomyopathy | Male | 7 mos | Mixed | Inconclusive |
| Case509 | HCM | HCM | Male | 62 yrs | White | Positive |
| Case510 | HCM | HCM | Unknown | 41 yrs | White | Negative |
| Case511 | LVNC | PanCardiomyopathy | Male | 41 yrs | Unspecified | Positive |
| Case512 | HCM | HCM | Male | 59 yrs | White | Negative |
| Case513 | HCM | HCM | Male | 26 yrs | White | Inconclusive |
| Case514 | HCM | HCM | Male | 19 yrs | White | Positive |
| Case515 | DCM | PanCardiomyopathy | Male | 54 yrs | White | Inconclusive |
| Case516 | DCM | PanCardiomyopathy | Male | 54 yrs | Hispanic or Latino | Positive |
| Case517 | Cardiomyopathy NOS | PanCardiomyopathy | Male | 3 yrs | Black or African American | Inconclusive |
| Case518 | HCM | HCM | Female | 52 yrs | White | Negative |
| Case519 | HCM | HCM | Male | 3 mos | White | Negative |
| Case520 | HCM | PanCardiomyopathy | Female | 45 yrs | White | Inconclusive |
| Case521 | HCM | PanCardiomyopathy | Male | 30 yrs | Unspecified | Positive |
| Case522 | HCM | PanCardiomyopathy | Female | 29 yrs | White | Positive |
| Case523 | DCM | PanCardiomyopathy | Female | 46 yrs | White | Inconclusive |
| Case524 | DCM | DCM | Female | 42 yrs | White | Negative |
| Case525 | HCM | HCM | Male | 35 yrs | Black or African American | Negative |
| Case526 | HCM | HCM | Male | 51 yrs | Black or African American | Negative |
| Case527 | HCM | HCM | Male | 66 yrs | White | Inconclusive |
| Case528 | Cardiomyopathy NOS | PanCardiomyopathy | Male | 2 mos | White | Negative |
| Case529 | ARVC | PanCardiomyopathy | Unknown | 48 yrs | Unspecified | Positive |
| Case530 | HCM | HCM | Male | 49 yrs | Unspecified | Positive |
| Case531 | HCM | HCM | Male | 28 yrs | White | Negative |
| Case532 | HCM | HCM | Female | 53 yrs | White | Inconclusive |
| Case533 | HCM | HCM | Male | 62 yrs | Unspecified | Negative |
| Case534 | HCM | PanCardiomyopathy | Male | 45 yrs | White | Inconclusive |
| Case535 | HCM | HCM | Female | 14 yrs | White | Negative |
| Case536 | HCM | PanCardiomyopathy | Male | 34 yrs | White | Positive |
| Case537 | HCM | HCM | Female | 84 yrs | White | Inconclusive |
| Case538 | HCM | PanCardiomyopathy | Female | 10 yrs | Mixed | Inconclusive |
| Case539 | HCM | HCM | Female | 70 yrs | White | Inconclusive |
| Case540 | HCM | PanCardiomyopathy | Female | 14 yrs | Black or African American | Positive |
| Case541 | HCM | PanCardiomyopathy | Male | 57 yrs | Unspecified | Inconclusive |
| Case542 | Cardiomyopathy NOS | PanCardiomyopathy | Female | 41 yrs | White | Inconclusive |
| Case543 | ARVC | ARVC | Male | 49 yrs | White | Inconclusive |
| Case544 | DCM | DCM | Male | 18 yrs | White | Positive |
| Case545 | Cardiomyopathy NOS | PanCardiomyopathy | Female | 6 yrs | Unspecified | Inconclusive |
| Case546 | DCM | DCM | Female | 45 yrs | White | Positive |
| Case547 | DCM | PanCardiomyopathy | Female | 2 mos | Unspecified | Inconclusive |
| Case548 | HCM | HCM | Male | 45 yrs | White | Negative |
| Case549 | HCM | HCM | Male | 54 yrs | Unspecified | Negative |
| Case550 | LVNC | PanCardiomyopathy | Male | 50 yrs | White | Negative |
| Case551 | DCM | PanCardiomyopathy | Male |  | White | Inconclusive |
| Case552 | HCM | HCM | Female | 28 yrs | White | Positive |
| Case553 | HCM | HCM | Male | 59 yrs | White | Inconclusive |
| Case554 | ARVC | ARVC | Male | 47 yrs | White | Negative |
| Case555 | DCM | PanCardiomyopathy | Unknown | 64 yrs | Unspecified | Inconclusive |
| Case556 | DCM | DCM | Male | 1 mos | Mixed | Positive |
| Case557 | HCM | HCM | Male | 57 yrs | White | Negative |
| Case558 | DCM | DCM | Female | 68 yrs | White | Positive |
| Case559 | DCM | PanCardiomyopathy | Male | 38 yrs | Unspecified | Positive |
| Case560 | DCM | PanCardiomyopathy | Male | 9 yrs | Mixed | Negative |
| Case561 | HCM | PanCardiomyopathy | Female | 15 yrs | White | Positive |
| Case562 | DCM | PanCardiomyopathy | Male | 20 yrs | White | Positive |
| Case563 | HCM | HCM | Male | 54 yrs | White | Negative |
| Case564 | RCM | PanCardiomyopathy | Female | 3 yrs | Asian | Inconclusive |
| Case565 | LVNC, HCM | PanCardiomyopathy | Male | 55 yrs | White | Positive |
| Case566 | HCM | HCM | Female | 46 yrs | White | Positive |
| Case567 | HCM | HCM | Male | 47 yrs | Unspecified | Negative |
| Case568 | HCM | HCM | Male | 44 yrs | White | Inconclusive |
| Case569 | DCM | DCM | Male | 14 yrs | Unspecified | Inconclusive |
| Case570 | DCM | DCM | Female | 1 mos | White | Inconclusive |
| Case571 | LVNC | PanCardiomyopathy | Female | 1 mos | Hispanic or Latino | Inconclusive |
| Case572 | HCM | HCM | Male | 54 yrs | White | Negative |
| Case573 | HCM | HCM | Male | 16 yrs | Black or African American | Inconclusive |
| Case574 | HCM | HCM | Female | 62 yrs | White | Inconclusive |
| Case575 | Cardiomyopathy NOS | PanCardiomyopathy | Female | 33 yrs | White | Inconclusive |
| Case576 | DCM | DCM | Female | 69 yrs | White | Negative |
| Case577 | HCM | HCM | Male | 55 yrs | White | Inconclusive |
| Case578 | DCM | DCM | Female | 53 yrs | White | Inconclusive |
| Case579 | HCM | HCM | Male | 53 yrs | White | Inconclusive |
| Case580 | HCM | HCM | Female | 72 yrs | White | Inconclusive |
| Case581 | HCM | HCM | Female | 34 yrs | White | Positive |
| Case582 | RCM | PanCardiomyopathy | Male | 10 mos | Unspecified | Inconclusive |
| Case583 | DCM | PanCardiomyopathy | Female | 44 yrs | White | Inconclusive |
| Case584 | HCM | HCM | Male | 43 yrs | White | Negative |
| Case585 | ARVC | ARVC | Female | 43 yrs | Unspecified | Negative |
| Case586 | HCM | HCM | Female | 87 yrs | White | Negative |
| Case587 | DCM | DCM | Female | 7 yrs | Unspecified | Inconclusive |
| Case588 | ARVC | PanCardiomyopathy | Male | 34 yrs | White | Negative |
| Case589 | LVNC | LVNC | Male | 1 yrs | American Indian or Alaska Native | Inconclusive |
| Case590 | DCM | DCM | Male | 1 yrs | Black or African American | Inconclusive |
| Case591 | HCM | HCM | Male | 15 yrs | Unspecified | Negative |
| Case592 | HCM | PanCardiomyopathy | Female | 66 yrs | Black or African American | Inconclusive |
| Case593 | ARVC | ARVC | Male | 24 yrs | White | Negative |
| Case594 | DCM | PanCardiomyopathy | Male | 15 yrs | Unspecified | Inconclusive |
| Case595 | HCM | HCM | Male | 17 yrs | Black or African American | Negative |
| Case596 | HCM | PanCardiomyopathy | Male | 36 yrs | White | Inconclusive |
| Case597 | HCM | PanCardiomyopathy | Male | 28 yrs | White | Positive |
| Case598 | ARVC | ARVC | Female | 56 yrs | Unspecified | Positive |
| Case599 | HCM | HCM | Male | 40 yrs | White | Negative |
| Case600 | DCM | PanCardiomyopathy | Male | 57 yrs | White | Inconclusive |
| Case601 | DCM | PanCardiomyopathy | Female | 44 yrs | White | Inconclusive |
| Case602 | DCM | PanCardiomyopathy | Female | 46 yrs | White | Inconclusive |
| Case603 | HCM | HCM | Female | 50 yrs | Unspecified | Negative |
| Case604 | HCM | PanCardiomyopathy | Female | 35 yrs | White | Inconclusive |
| Case605 | HCM | PanCardiomyopathy | Male | 55 yrs | Ashkenazi Jewish | Inconclusive |
| Case606 | HCM | HCM | Female | 86 yrs | White | Negative |
| Case607 | DCM | PanCardiomyopathy | Male |  | Black or African American | Inconclusive |
| Case608 | HCM | PanCardiomyopathy | Unknown | 12 yrs | White | Inconclusive |
| Case609 | DCM | PanCardiomyopathy | Female | 9 yrs | White | Inconclusive |
| Case610 | HCM | HCM | Female | 61 yrs | Black or African American | Negative |
| Case611 | DCM | PanCardiomyopathy | Female | 15 yrs | Unspecified | Inconclusive |
| Case612 | DCM | PanCardiomyopathy | Female | 14 yrs | White | Inconclusive |
| Case613 | HCM | HCM | Male | 57 yrs | White | Positive |
| Case614 | HCM | PanCardiomyopathy | Male | 53 yrs | White | Positive |
| Case615 | DCM | PanCardiomyopathy | Male | 11 yrs | Mixed | Positive |
| Case616 | HCM | HCM | Male | 18 yrs | Black or African American | Negative |
| Case617 | HCM | HCM | Male | 65 yrs | White | Negative |
| Case618 | HCM | HCM | Male | 70 yrs | White | Negative |
| Case619 | HCM | HCM | Female | 69 yrs | Asian | Negative |
| Case620 | HCM | HCM | Female | 47 yrs | Black or African American | Negative |
| Case621 | DCM | PanCardiomyopathy | Male | 17 yrs | White | Negative |
| Case622 | DCM | PanCardiomyopathy | Female | 12 yrs | Unspecified | Inconclusive |
| Case623 | DCM | DCM | Male | 18 yrs | Hispanic or Latino | Inconclusive |
| Case624 | HCM | HCM | Female | 69 yrs | White | Negative |
| Case625 | HCM | HCM | Male | 57 yrs | White | Positive |
| Case626 | ARVC | ARVC | Male | 29 yrs | White | Negative |
| Case627 | DCM | PanCardiomyopathy | Female | 9 mos | White | Positive |
| Case628 | ARVC | ARVC | Female | 74 yrs | Unspecified | Negative |
| Case629 | HCM | HCM | Female | 34 yrs | White | Negative |
| Case630 | HCM | HCM | Male | 65 yrs | White | Negative |
| Case631 | HCM | HCM | Male | 28 yrs | White | Positive |
| Case632 | HCM | HCM | Male | 70 yrs | White | Negative |
| Case633 | HCM | HCM | Male | 65 yrs | White | Negative |
| Case634 | DCM | PanCardiomyopathy | Male | 1 mos | White | Inconclusive |
| Case635 | HCM | HCM | Female | 52 yrs | Unspecified | Inconclusive |
| Case636 | Cardiomyopathy NOS | PanCardiomyopathy | Female | 17 yrs | Mixed | Inconclusive |
| Case637 | HCM | HCM | Female | 45 yrs | White | Negative |
| Case638 | HCM | PanCardiomyopathy | Unknown | 26 yrs | Black or African American | Inconclusive |
| Case639 | HCM | PanCardiomyopathy | Male | 20 yrs | Black or African American | Inconclusive |
| Case640 | DCM | DCM | Female |  | Mixed | Inconclusive |
| Case641 | HCM | PanCardiomyopathy | Male | 32 yrs | White | Inconclusive |
| Case642 | HCM | HCM | Male | 9 mos | White | Negative |
| Case643 | DCM | PanCardiomyopathy | Male | 61 yrs | White | Inconclusive |
| Case644 | DCM | PanCardiomyopathy | Female | 1 yrs | Black or African American | Inconclusive |
| Case645 | HCM | PanCardiomyopathy | Male | 62 yrs | Unspecified | Positive |
| Case646 | DCM | DCM | Male | 13 yrs | Unspecified | Inconclusive |
| Case647 | DCM | PanCardiomyopathy | Unknown | 1 yrs | Hispanic or Latino | Inconclusive |
| Case648 | DCM | PanCardiomyopathy | Female | 60 yrs | White | Inconclusive |
| Case649 | DCM | PanCardiomyopathy | Unknown | 58 yrs | Unspecified | Negative |
| Case650 | ARVC | PanCardiomyopathy | Female | 22 yrs | White | Inconclusive |
| Case651 | HCM | PanCardiomyopathy | Male | 44 yrs | Unspecified | Inconclusive |
| Case652 | Cardiomyopathy NOS | PanCardiomyopathy | Female | 18 yrs | White | Inconclusive |
| Case653 | HCM | HCM | Female | 64 yrs | White | Positive |
| Case654 | HCM | HCM | Male | 27 yrs | White | Negative |
| Case655 | DCM | PanCardiomyopathy | Female | 42 yrs | White | Inconclusive |
| Case656 | DCM | PanCardiomyopathy | Male | 25 yrs | White | Inconclusive |
| Case657 | DCM | PanCardiomyopathy | Female | 15 yrs | White | Inconclusive |
| Case658 | DCM | PanCardiomyopathy | Male | 25 yrs | Unspecified | Inconclusive |
| Case659 | HCM | PanCardiomyopathy | Male | 64 yrs | Asian | Positive |
| Case660 | LVNC, HCM | PanCardiomyopathy | Male | 1 mos | Hispanic or Latino | Inconclusive |
| Case661 | HCM | HCM | Male | 55 yrs | White | Inconclusive |
| Case662 | ARVC | ARVC | Male | 58 yrs | White | Positive |
| Case663 | DCM | PanCardiomyopathy | Female | 56 yrs | White | Negative |
| Case664 | HCM | HCM | Female | 4 mos | Unspecified | Negative |
| Case665 | HCM | HCM | Female | 71 yrs | White | Negative |
| Case666 | DCM | PanCardiomyopathy | Male | 21 yrs | Black or African American | Positive |
| Case667 | ARVC | ARVC | Female | 57 yrs | White | Positive |
| Case668 | HCM | HCM | Female | 26 yrs | Unspecified | Negative |
| Case669 | HCM | HCM | Female | 41 yrs | Unspecified | Negative |
| Case670 | DCM | DCM | Female | 6 yrs | Unspecified | Inconclusive |
| Case671 | DCM | PanCardiomyopathy | Male | 3 mos | Unspecified | Inconclusive |
| Case672 | HCM | HCM | Male | 63 yrs | White | Inconclusive |
| Case673 | HCM | PanCardiomyopathy | Male | 58 yrs | Black or African American | Inconclusive |
| Case674 | HCM | PanCardiomyopathy | Male | 26 yrs | Unspecified | Positive |
| Case675 | HCM | HCM | Female | 73 yrs | White | Negative |
| Case676 | HCM | HCM | Male | 40 yrs | White | Negative |
| Case677 | HCM | HCM | Female | 56 yrs | White | Positive |
| Case678 | HCM | HCM | Male | 24 yrs | White | Positive |
| Case679 | DCM | PanCardiomyopathy | Female | 30 yrs | American Indian or Alaska Native | Positive |
| Case680 | DCM | PanCardiomyopathy | Male | 4 mos | Unspecified | Inconclusive |
| Case681 | HCM | HCM | Male | 54 yrs | White | Negative |
| Case682 | DCM | PanCardiomyopathy | Male | 15 yrs | Unspecified | Positive |
| Case683 | LVNC | PanCardiomyopathy | Female | 24 yrs | White | Negative |
| Case684 | HCM | PanCardiomyopathy | Male | 3 mos | Unspecified | Inconclusive |
| Case685 | ARVC | PanCardiomyopathy | Female | 43 yrs | White | Positive |
| Case686 | HCM | PanCardiomyopathy | Female | 58 yrs | White | Positive |
| Case687 | RCM | PanCardiomyopathy | Male | 11 yrs | Black or African American | Positive |
| Case688 | DCM | PanCardiomyopathy | Male | 61 yrs | White | Positive |
| Case689 | DCM | PanCardiomyopathy | Female | 6 mos | Unspecified | Inconclusive |
| Case690 | DCM, Myopathy | PanCardiomyopathy | Female | 13 yrs | Mixed | Positive |
| Case691 | DCM | PanCardiomyopathy | Female | 43 yrs | White | Inconclusive |
| Case692 | DCM | PanCardiomyopathy | Male | 41 yrs | White | Positive |
| Case693 | DCM | PanCardiomyopathy | Female | 15 yrs | White | Positive |
| Case694 | HCM | PanCardiomyopathy | Male | 35 yrs | Asian | Positive |
| Case695 | DCM | PanCardiomyopathy | Male | 19 yrs | White | Negative |
| Case696 | DCM | PanCardiomyopathy | Female |  | Mixed | Inconclusive |
| Case697 | DCM | PanCardiomyopathy | Female | 30 yrs | White | Positive |
| Case698 | RCM | PanCardiomyopathy | Male | 16 yrs | White | Positive |
| Case699 | DCM | PanCardiomyopathy | Male | 9 mos | Unspecified | Negative |
| Case700 | HCM | HCM | Male | 32 yrs | Unspecified | Positive |
| Case701 | DCM | PanCardiomyopathy | Female | 10 mos | Black or African American | Inconclusive |
| Case702 | HCM | HCM | Female | 14 yrs | Unspecified | Positive |
| Case703 | DCM | PanCardiomyopathy | Male | 19 yrs | White | Inconclusive |
| Case704 | DCM | PanCardiomyopathy | Male | 31 yrs | White | Inconclusive |
| Case705 | HCM | HCM | Female | 43 yrs | White | Negative |
| Case706 | HCM | HCM | Female | 48 yrs | White | Negative |
| Case707 | HCM | HCM | Male | 78 yrs | Unspecified | Negative |
| Case708 | HCM | PanCardiomyopathy | Male | 44 yrs | Unspecified | Positive |
| Case709 | HCM | PanCardiomyopathy | Male | 44 yrs | Black or African American | Inconclusive |
| Case710 | DCM | PanCardiomyopathy | Female | 6 mos | White | Inconclusive |
| Case711 | DCM | PanCardiomyopathy | Female | 13 yrs | White | Inconclusive |
| Case712 | HCM | HCM | Female | 65 yrs | Unspecified | Negative |
| Case713 | DCM | PanCardiomyopathy | Male | 31 yrs | Unspecified | Inconclusive |
| Case714 | DCM | PanCardiomyopathy | Male | 7 yrs | Mixed | Inconclusive |
| Case715 | ARVC | ARVC | Male | 49 yrs | White | Inconclusive |
| Case716 | DCM | PanCardiomyopathy | Male | 14 yrs | White | Inconclusive |
| Case717 | DCM | PanCardiomyopathy | Male | 31 yrs | Unspecified | Positive |
| Case718 | DCM | DCM | Male | 9 yrs | Unspecified | Inconclusive |
| Case719 | HCM | PanCardiomyopathy | Male | 18 yrs | Asian | Inconclusive |
| Case720 | HCM | PanCardiomyopathy | Male | 32 yrs | Mixed | Inconclusive |
| Case721 | HCM | HCM | Female | 69 yrs | American Indian or Alaska Native | Inconclusive |
| Case722 | HCM | HCM | Male | 65 yrs | Unspecified | Positive |
| Case723 | LVNC | PanCardiomyopathy | Male | 1 mos | White | Inconclusive |
| Case724 | DCM | DCM | Female | 55 yrs | White | Inconclusive |
| Case725 | HCM | HCM | Male | 69 yrs | White | Inconclusive |
| Case726 | LVNC | PanCardiomyopathy | Male | 19 yrs | White | Inconclusive |
| Case727 | HCM | HCM | Female | 57 yrs | White | Negative |
| Case728 | HCM | HCM | Female | 80 yrs | Unspecified | Inconclusive |
| Case729 | HCM | PanCardiomyopathy | Male | 38 yrs | Unspecified | Inconclusive |
| Case730 | DCM | PanCardiomyopathy | Male | 23 yrs | White | Inconclusive |
| Case731 | HCM | HCM | Male | 57 yrs | Unspecified | Inconclusive |
| Case732 | DCM | PanCardiomyopathy | Unknown | 55 yrs | Unspecified | Inconclusive |
| Case733 | LVNC | PanCardiomyopathy | Female |  | Unspecified | Inconclusive |
| Case734 | HCM | HCM | Male | 10 yrs | Asian | Positive |
| Case735 | HCM | HCM | Male | 44 yrs | White | Negative |
| Case736 | HCM | HCM | Male | 34 yrs | White | Positive |
| Case737 | DCM | DCM | Female | 48 yrs | Mixed | Inconclusive |
| Case738 | DCM | PanCardiomyopathy | Female | 49 yrs | Unspecified | Positive |
| Case739 | HCM | HCM | Female | 2 yrs | White | Negative |
| Case740 | HCM | HCM | Male | 45 yrs | White | Negative |
| Case741 | ARVC | PanCardiomyopathy | Female | 30 yrs | White | Positive |
| Case742 | HCM | HCM | Unknown | 8 yrs | White | Negative |
| Case743 | DCM | PanCardiomyopathy | Female | 15 yrs | White | Inconclusive |
| Case744 | HCM | HCM | Male | 11 yrs | Ashkenazi Jewish | Positive |
| Case745 | HCM | PanCardiomyopathy | Male | 8 mos | Mixed | Inconclusive |
| Case746 | HCM | HCM | Female | 63 yrs | White | Positive |
| Case747 | ARVC | ARVC | Male | 29 yrs | Unspecified | Inconclusive |
| Case748 | DCM | PanCardiomyopathy | Male | 7 mos | White | Inconclusive |
| Case749 | DCM | PanCardiomyopathy | Female | 10 yrs | Unspecified | Positive |
| Case750 | HCM | HCM | Female | 82 yrs | Asian | Positive |
| Case751 | Cardiomyopathy NOS | PanCardiomyopathy | Female | 1 mos | Unspecified | Inconclusive |
| Case752 | DCM | PanCardiomyopathy | Female | 37 yrs | White | Inconclusive |
| Case753 | Cardiomyopathy NOS | PanCardiomyopathy | Male | 13 yrs | Black or African American | Inconclusive |
| Case754 | HCM | PanCardiomyopathy | Female | 52 yrs | Unspecified | Positive |
| Case755 | HCM | PanCardiomyopathy | Male | 44 yrs | White | Inconclusive |
| Case756 | HCM | HCM | Male | 64 yrs | White | Inconclusive |
| Case757 | HCM | HCM | Male | 56 yrs | White | Negative |
| Case758 | HCM | HCM | Female | 66 yrs | White | Negative |
| Case759 | HCM | HCM | Female | 27 yrs | White | Negative |
| Case760 | HCM | HCM | Male | 18 yrs | White | Positive |
| Case761 | HCM | HCM | Male | 63 yrs | White | Negative |
| Case762 | HCM | HCM | Male | 37 yrs | White | Positive |
| Case763 | HCM | HCM | Male | 61 yrs | White | Negative |
| Case764 | HCM | HCM | Male | 29 yrs | White | Inconclusive |
| Case765 | HCM | HCM | Male | 56 yrs | White | Negative |
| Case766 | DCM | PanCardiomyopathy | Female | 32 yrs | White | Inconclusive |
| Case767 | HCM | HCM | Male | 27 yrs | White | Negative |
| Case768 | DCM | PanCardiomyopathy | Male | 10 mos | Asian | Inconclusive |
| Case769 | DCM | PanCardiomyopathy | Female | 1 yrs | Asian | Inconclusive |
| Case770 | Cardiomyopathy NOS | PanCardiomyopathy | Male | 17 yrs | White | Inconclusive |
| Case771 | HCM | PanCardiomyopathy | Male | 48 yrs | Mixed | Negative |
| Case772 | HCM | HCM | Female | 30 yrs | Black or African American | Negative |
| Case773 | HCM | HCM | Male | 77 yrs | Unspecified | Positive |
| Case774 | HCM | HCM | Female | 28 yrs | Black or African American | Inconclusive |
| Case775 | Cardiomyopathy NOS | PanCardiomyopathy | Male | 62 yrs | White | Positive |
| Case776 | HCM | PanCardiomyopathy | Female | 7 mos | Black or African American | Inconclusive |
| Case777 | HCM | HCM | Female | 59 yrs | White | Positive |
| Case778 | HCM | HCM | Male | 50 yrs | Unspecified | Positive |
| Case779 | HCM | HCM | Female | 62 yrs | Black or African American | Negative |
| Case780 | RCM | PanCardiomyopathy | Female | 2 yrs | Unspecified | Positive |
| Case781 | DCM | PanCardiomyopathy | Female | 20 yrs | White | Positive |
| Case782 | HCM | HCM | Female | 65 yrs | Mixed | Negative |
| Case783 | HCM | PanCardiomyopathy | Male | 20 yrs | White | Negative |
| Case784 | DCM | PanCardiomyopathy | Male | 61 yrs | White | Positive |
| Case785 | DCM | PanCardiomyopathy | Male | 65 yrs | White | Inconclusive |
| Case786 | DCM | DCM | Female | 9 mos | Hispanic or Latino | Inconclusive |
| Case787 | HCM | HCM | Female | 66 yrs | White | Positive |
| Case788 | DCM | PanCardiomyopathy | Male | 48 yrs | White | Inconclusive |
| Case789 | DCM | PanCardiomyopathy | Male | 2 mos | Unspecified | Inconclusive |
| Case790 | DCM | PanCardiomyopathy | Female | 76 yrs | Unspecified | Inconclusive |
| Case791 | HCM | HCM | Female | 72 yrs | Unspecified | Negative |
| Case792 | HCM | HCM | Male | 37 yrs | White | Negative |
| Case793 | DCM | PanCardiomyopathy | Male | 17 yrs | White | Positive |
| Case794 | DCM | PanCardiomyopathy | Male | 19 yrs | White | Inconclusive |
| Case795 | HCM | PanCardiomyopathy | Male | 12 yrs | Unspecified | Inconclusive |
| Case796 | HCM | HCM | Female | 84 yrs | White | Inconclusive |
| Case797 | HCM | HCM | Male | 74 yrs | Unspecified | Negative |
| Case798 | DCM | PanCardiomyopathy | Male | 43 yrs | White | Inconclusive |
| Case799 | DCM | PanCardiomyopathy | Male | 65 yrs | White | Positive |
| Case800 | HCM | PanCardiomyopathy | Male | 64 yrs | Ashkenazi Jewish | Inconclusive |
| Case801 | DCM | PanCardiomyopathy | Male | 8 yrs | White | Inconclusive |
| Case802 | Cardiomyopathy NOS | PanCardiomyopathy | Female | 41 yrs | White | Inconclusive |
| Case803 | DCM | DCM | Male | 16 yrs | Unspecified | Inconclusive |
| Case804 | DCM | PanCardiomyopathy | Male | 22 yrs | White | Positive |
| Case805 | DCM | PanCardiomyopathy | Female | 54 yrs | White | Inconclusive |
| Case806 | DCM | PanCardiomyopathy | Female | 48 yrs | White | Positive |
| Case807 | DCM | PanCardiomyopathy | Female | 54 yrs | White | Inconclusive |
| Case808 | HCM | PanCardiomyopathy | Male | 65 yrs | White | Inconclusive |
| Case809 | LVNC | PanCardiomyopathy | Female | 1 mos | Black or African American | Negative |
| Case810 | HCM | PanCardiomyopathy | Male | 18 yrs | White | Inconclusive |
| Case811 | DCM | DCM | Male | 18 yrs | White | Inconclusive |
| Case812 | DCM | DCM | Female | 56 yrs | White | Negative |
| Case813 | LVNC | LVNC | Male | 10 yrs | White | Inconclusive |
| Case814 | HCM | HCM | Male | 82 yrs | White | Negative |
| Case815 | HCM | HCM | Male | 50 yrs | Unspecified | Negative |
| Case816 | HCM | HCM | Female | 30 yrs | White | Positive |
| Case817 | DCM | PanCardiomyopathy | Male |  | Unspecified | Inconclusive |
| Case818 | LVNC | PanCardiomyopathy | Male | 6 yrs | Unspecified | Inconclusive |
| Case819 | DCM | DCM | Male | 60 yrs | White | Positive |
| Case820 | DCM | DCM | Female | 43 yrs | White | Positive |
| Case821 | DCM | PanCardiomyopathy | Female | 19 yrs | White | Inconclusive |
| Case822 | HCM | HCM | Female | 12 yrs | White | Inconclusive |
| Case823 | HCM | HCM | Male | 67 yrs | Mixed | Negative |
| Case824 | HCM | HCM | Male | 29 yrs | Unspecified | Negative |
| Case825 | DCM | PanCardiomyopathy | Male | 48 yrs | White | Inconclusive |
| Case826 | HCM | PanCardiomyopathy | Male | 51 yrs | White | Negative |
| Case827 | HCM | PanCardiomyopathy | Male | 31 yrs | Asian | Inconclusive |
| Case828 | HCM | HCM | Female | 46 yrs | White | Negative |
| Case829 | HCM | HCM | Female | 68 yrs | White | Negative |
| Case830 | ARVC | PanCardiomyopathy | Female | 39 yrs | White | Inconclusive |
| Case831 | HCM | HCM | Male | 2 mos | Hispanic or Latino | Negative |
| Case832 | HCM, LVNC | PanCardiomyopathy | Male | 1 yrs | Ashkenazi Jewish | Inconclusive |
| Case833 | DCM | DCM | Male | 3 mos | White | Inconclusive |
| Case834 | DCM | PanCardiomyopathy | Male | 10 mos | Mixed | Inconclusive |
| Case835 | DCM | DCM | Male | 60 yrs | White | Inconclusive |
| Case836 | HCM | HCM | Female | 65 yrs | White | Negative |
| Case837 | HCM | HCM | Male | 54 yrs | White | Negative |
| Case838 | HCM | PanCardiomyopathy | Male | 46 yrs | White | Inconclusive |
| Case839 | HCM | HCM | Female | 80 yrs | White | Negative |
| Case840 | ARVC | PanCardiomyopathy | Male | 52 yrs | White | Inconclusive |
| Case841 | HCM | PanCardiomyopathy | Male | 53 yrs | White | Inconclusive |
| Case842 | LVNC | LVNC | Male | 9 yrs | White | Negative |
| Case843 | DCM | PanCardiomyopathy | Female | 48 yrs | White | Positive |
| Case844 | HCM | PanCardiomyopathy | Male | 26 yrs | White | Inconclusive |
| Case845 | DCM | PanCardiomyopathy | Female | 53 yrs | Unspecified | Positive |
| Case846 | ARVC | ARVC | Male | 13 yrs | White | Positive |
| Case847 | HCM | PanCardiomyopathy | Male | 15 yrs | Unspecified | Inconclusive |
| Case848 | DCM, HCM | PanCardiomyopathy | Female |  | White | Inconclusive |
| Case849 | DCM | PanCardiomyopathy | Male | 59 yrs | White | Negative |
| Case850 | HCM | HCM | Male | 21 yrs | Black or African American | Negative |
| Case851 | HCM | HCM | Male | 55 yrs | White | Negative |
| Case852 | DCM | PanCardiomyopathy | Female | 6 mos | White | Inconclusive |
| Case853 | DCM | PanCardiomyopathy | Female | 3 mos | White | Inconclusive |
| Case854 | HCM | HCM | Female | 15 yrs | Unspecified | Negative |
| Case855 | ARVC | PanCardiomyopathy | Female | 53 yrs | Unspecified | Inconclusive |
| Case856 | HCM | PanCardiomyopathy | Male | 51 yrs | Mixed | Positive |
| Case857 | DCM | PanCardiomyopathy | Female | 10 yrs | White | Inconclusive |
| Case858 | RCM | PanCardiomyopathy | Female | 1 mos | Mixed | Inconclusive |
| Case859 | DCM, ARVC | PanCardiomyopathy | Female | 21 yrs | White | Inconclusive |
| Case860 | ARVC | PanCardiomyopathy | Male | 7 yrs | Mixed | Inconclusive |
| Case861 | DCM | PanCardiomyopathy | Male | 32 yrs | Unspecified | Inconclusive |
| Case862 | DCM | PanCardiomyopathy | Female | 30 yrs | Unspecified | Inconclusive |
| Case863 | DCM | PanCardiomyopathy | Female | 2 yrs | Ashkenazi Jewish | Inconclusive |
| Case864 | HCM | PanCardiomyopathy | Male | 2 yrs | Black or African American | Inconclusive |
| Case865 | HCM | PanCardiomyopathy | Male | 48 yrs | Ashkenazi Jewish | Inconclusive |
| Case866 | DCM, LVNC | PanCardiomyopathy | Female | 11 yrs | Mixed | Positive |
| Case867 | HCM | HCM | Male | 40 yrs | Asian | Negative |
| Case868 | DCM | PanCardiomyopathy | Female | 17 yrs | Unspecified | Inconclusive |
| Case869 | HCM | HCM | Female | 84 yrs | Unspecified | Inconclusive |
| Case870 | HCM | HCM | Female | 22 yrs | White | Positive |
| Case871 | DCM | PanCardiomyopathy | Male | 4 mos | Asian | Positive |
| Case872 | DCM | DCM | Male | 1 mos | Unspecified | Inconclusive |
| Case873 | HCM | PanCardiomyopathy | Male | 9 yrs | White | Inconclusive |
| Case874 | LVNC | PanCardiomyopathy | Female | 11 yrs | Hispanic or Latino | Negative |
| Case875 | HCM | HCM | Female | 70 yrs | White | Negative |
| Case876 | ARVC | PanCardiomyopathy | Male | 67 yrs | White | Inconclusive |
| Case877 | HCM | PanCardiomyopathy | Male | 64 yrs | Ashkenazi Jewish | Inconclusive |
| Case878 | DCM | PanCardiomyopathy | Female | 21 yrs | White | Inconclusive |
| Case879 | HCM | PanCardiomyopathy | Female | 52 yrs | White | Positive |
| Case880 | HCM | PanCardiomyopathy | Female | 80 yrs | Unspecified | Positive |
| Case881 | HCM | HCM | Female | 84 yrs | Unspecified | Negative |
| Case882 | HCM | PanCardiomyopathy | Unknown | 64 yrs | White | Inconclusive |
| Case883 | HCM | HCM | Male | 65 yrs | White | Inconclusive |
| Case884 | HCM | PanCardiomyopathy | Female | 62 yrs | White | Positive |
| Case885 | HCM | PanCardiomyopathy | Male | 16 yrs | Black or African American | Inconclusive |
| Case886 | HCM | PanCardiomyopathy | Male | 43 yrs | White | Inconclusive |
| Case887 | DCM, LVNC | PanCardiomyopathy | Female | 25 yrs | Black or African American | Inconclusive |
| Case888 | DCM, LVNC | PanCardiomyopathy | Female | 15 yrs | White | Positive |
| Case889 | HCM | PanCardiomyopathy | Male | 10 yrs | White | Negative |
| Case890 | DCM | PanCardiomyopathy | Female | 1 mos | Unspecified | Negative |
| Case891 | HCM | PanCardiomyopathy | Male | 9 yrs | White | Inconclusive |
| Case892 | ARVC | ARVC | Male | 8 yrs | White | Negative |
| Case893 | HCM | PanCardiomyopathy | Male | 3 mos | Hispanic or Latino | Inconclusive |
| Case894 | HCM | PanCardiomyopathy | Female | 48 yrs | White | Inconclusive |
| Case895 | HCM | HCM | Female | 78 yrs | Unspecified | Negative |
| Case896 | DCM | DCM | Female | 39 yrs | Unspecified | Inconclusive |
| Case897 | DCM | DCM | Male | 2 mos | Hispanic or Latino | Inconclusive |
| Case898 | LVNC | PanCardiomyopathy | Male | 6 mos | Black or African American | Inconclusive |
| Case899 | HCM | HCM | Male | 2 mos | White | Inconclusive |
| Case900 | LVNC | LVNC | Female | 37 yrs | Unspecified | Inconclusive |
| Case901 | DCM | DCM | Male | 66 yrs | White | Positive |
| Case902 | RCM | PanCardiomyopathy | Female | 2 yrs | White | Inconclusive |
| Case903 | DCM | PanCardiomyopathy | Female | 25 yrs | White | Positive |
| Case904 | DCM | PanCardiomyopathy | Male | 3 yrs | Unspecified | Inconclusive |
| Case905 | HCM | PanCardiomyopathy | Male | 15 yrs | Unspecified | Inconclusive |
| Case906 | HCM | HCM | Female | 47 yrs | White | Positive |
| Case907 | HCM | HCM | Male | 71 yrs | White | Positive |
| Case908 | DCM | DCM | Female | 59 yrs | White | Inconclusive |
| Case909 | DCM | PanCardiomyopathy | Female | 54 yrs | White | Positive |
| Case910 | HCM | HCM | Male | 47 yrs | Unspecified | Positive |
| Case911 | RCM | PanCardiomyopathy | Male | 2 yrs | Unspecified | Inconclusive |
| Case912 | HCM | HCM | Female | 68 yrs | Unspecified | Negative |
| Case913 | DCM | PanCardiomyopathy | Male | 2 mos | White | Inconclusive |
| Case914 | HCM | PanCardiomyopathy | Male | 58 yrs | White | Inconclusive |
| Case915 | DCM, ARVC | PanCardiomyopathy | Male | 58 yrs | White | Positive |
| Case916 | HCM | HCM | Male | 17 yrs | White | Negative |
| Case917 | HCM | PanCardiomyopathy | Female | 16 yrs | Black or African American | Inconclusive |
| Case918 | DCM | DCM | Female | 39 yrs | White | Inconclusive |
| Case919 | HCM | HCM | Male | 47 yrs | White | Negative |
| Case920 | HCM | HCM | Male | 29 yrs | White | Positive |
| Case921 | HCM | HCM | Male | 58 yrs | White | Positive |
| Case922 | DCM | DCM | Female | 51 yrs | White | Negative |
| Case923 | DCM | PanCardiomyopathy | Female | 53 yrs | White | Inconclusive |
| Case924 | HCM | PanCardiomyopathy | Male | 57 yrs | Unspecified | Inconclusive |
| Case925 | HCM | PanCardiomyopathy | Female | 63 yrs | White | Inconclusive |
| Case926 | LVNC | PanCardiomyopathy | Female | 62 yrs | Unspecified | Positive |
| Case927 | HCM | HCM | Male | 19 yrs | Unspecified | Inconclusive |
| Case928 | HCM | PanCardiomyopathy | Female | 60 yrs | White | Inconclusive |
| Case929 | DCM | PanCardiomyopathy | Female | 47 yrs | Hispanic or Latino | Inconclusive |
| Case930 | HCM | HCM | Male | 60 yrs | White | Negative |
| Case931 | Cardiomyopathy NOS | HCM | Male | 20 yrs | White | Negative |
| Case932 | HCM | HCM | Female | 58 yrs | White | Negative |
| Case933 | HCM | HCM | Male | 52 yrs | White | Negative |
| Case934 | DCM | PanCardiomyopathy | Male | 17 yrs | White | Inconclusive |
| Case935 | HCM | HCM | Male | 53 yrs | White | Negative |
| Case936 | HCM | PanCardiomyopathy | Female | 56 yrs | White | Inconclusive |
| Case937 | DCM | PanCardiomyopathy | Female | 36 yrs | Hispanic or Latino | Inconclusive |
| Case938 | HCM | HCM | Female | 14 yrs | White | Positive |
| Case939 | DCM | DCM | Female | 58 yrs | White | Negative |
| Case940 | HCM | HCM | Male | 55 yrs | White | Negative |
| Case941 | DCM | PanCardiomyopathy | Male | 29 yrs | Unspecified | Inconclusive |
| Case942 | DCM | PanCardiomyopathy | Female | 37 yrs | White | Positive |
| Case943 | HCM | HCM | Female | 61 yrs | Hispanic or Latino | Negative |
| Case944 | DCM | PanCardiomyopathy | Male | 51 yrs | White | Inconclusive |
| Case945 | HCM | PanCardiomyopathy | Male | 48 yrs | Unspecified | Inconclusive |
| Case946 | Cardiomyopathy NOS, Myopathy | PanCardiomyopathy | Male | 53 yrs | White | Inconclusive |
| Case947 | DCM | PanCardiomyopathy | Male | 63 yrs | White | Inconclusive |
| Case948 | HCM | HCM | Male |  | White | Positive |
| Case949 | HCM | PanCardiomyopathy | Female | 7 mos | White | Positive |
| Case950 | ARVC | PanCardiomyopathy | Male | 49 yrs | White | Inconclusive |
| Case951 | DCM | PanCardiomyopathy | Male | 27 yrs | White | Inconclusive |
| Case952 | DCM | PanCardiomyopathy | Male | 7 yrs | White | Inconclusive |
| Case953 | HCM | PanCardiomyopathy | Male | 61 yrs | White | Inconclusive |
| Case954 | DCM | DCM | Female | 13 yrs | White | Inconclusive |
| Case955 | HCM | HCM | Female | 19 yrs | White | Positive |
| Case956 | HCM | HCM | Male | 61 yrs | Black or African American | Negative |
| Case957 | ARVC | ARVC | Male | 39 yrs | White | Negative |
| Case958 | HCM | PanCardiomyopathy | Male | 57 yrs | Unspecified | Inconclusive |
| Case959 | HCM | PanCardiomyopathy | Female | 62 yrs | White | Negative |
| Case960 | DCM | PanCardiomyopathy | Female | 1 yrs | Hispanic or Latino | Inconclusive |
| Case961 | HCM | HCM | Female | 52 yrs | Unspecified | Positive |
| Case962 | HCM | HCM | Female | 27 yrs | Black or African American | Negative |
| Case963 | HCM | HCM | Male | 16 yrs | Hispanic or Latino | Positive |
| Case964 | HCM | HCM | Female | 64 yrs | Black or African American | Negative |
| Case965 | HCM | HCM | Male | 70 yrs | Ashkenazi Jewish | Negative |
| Case966 | HCM | PanCardiomyopathy | Male | 43 yrs | Black or African American | Inconclusive |
| Case967 | LVNC | PanCardiomyopathy | Male | 31 yrs | Unspecified | Inconclusive |
| Case968 | HCM | HCM | Male | 45 yrs | White | Negative |
| Case969 | HCM | HCM | Female | 44 yrs | White | Negative |
| Case970 | HCM | PanCardiomyopathy | Male | 9 mos | Unspecified | Inconclusive |
| Case971 | HCM | HCM | Female | 62 yrs | White | Negative |
| Case972 | DCM | DCM | Unspecified | 65 yrs | White | Positive |
| Case973 | ARVC | ARVC | Male | 57 yrs | White | Negative |
| Case974 | HCM | PanCardiomyopathy | Female | 3 yrs | Unspecified | Positive |
| Case975 | Cardiomyopathy NOS, Myopathy | PanCardiomyopathy | Male | 6 mos | Unspecified | Inconclusive |
| Case976 | HCM | HCM | Female | 59 yrs | Black or African American | Negative |
| Case977 | HCM | HCM | Male | 38 yrs | Unspecified | Inconclusive |
| Case978 | DCM | PanCardiomyopathy | Male | 63 yrs | White | Positive |
| Case979 | HCM | HCM | Female | 33 yrs | Unspecified | Positive |
| Case980 | RCM, LVNC | PanCardiomyopathy | Male | 14 yrs | Black or African American | Inconclusive |
| Case981 | DCM | PanCardiomyopathy | Male | 20 yrs | White | Inconclusive |
| Case982 | HCM | HCM | Male | 20 yrs | White | Inconclusive |
| Case983 | ARVC | PanCardiomyopathy | Female | 64 yrs | Unspecified | Inconclusive |
| Case984 | RCM | PanCardiomyopathy | Female | 33 yrs | White | Positive |
| Case985 | HCM | HCM | Male | 72 yrs | White | Negative |
| Case986 | HCM | HCM | Female | 14 yrs | White | Inconclusive |
| Case987 | DCM | DCM | Male | 21 yrs | White | Inconclusive |
| Case988 | DCM | DCM | Male | 15 yrs | White | Inconclusive |
| Case989 | DCM | PanCardiomyopathy | Male | 48 yrs | Black or African American | Negative |
| Case990 | DCM | DCM | Male | 54 yrs | Black or African American | Positive |
| Case991 | HCM | HCM | Female | 50 yrs | Mixed | Negative |
| Case992 | HCM | PanCardiomyopathy | Male | 58 yrs | White | Inconclusive |
| Case993 | DCM | PanCardiomyopathy | Female | 32 yrs | White | Inconclusive |
| Case994 | DCM | PanCardiomyopathy | Female | 8 mos | Black or African American | Inconclusive |
| Case995 | HCM | HCM | Female | 59 yrs | Unspecified | Inconclusive |
| Case996 | HCM | HCM | Female | 82 yrs | White | Negative |
| Case997 | DCM | PanCardiomyopathy | Female | 66 yrs | Unspecified | Inconclusive |
| Case998 | ARVC | ARVC | Male | 63 yrs | White | Inconclusive |
| Case999 | DCM | PanCardiomyopathy | Female | 49 yrs | White | Inconclusive |
| Case1000 | RCM | PanCardiomyopathy | Male | 10 yrs | Black or African American | Positive |
| Case1001 | HCM | PanCardiomyopathy | Male | 63 yrs | White | Inconclusive |
| Case1002 | HCM | HCM | Female | 27 yrs | Unspecified | Positive |
| Case1003 | DCM | DCM | Female | 69 yrs | White | Inconclusive |
| Case1004 | HCM | HCM | Female | 76 yrs | White | Positive |
| Case1005 | HCM | HCM | Male | 16 yrs | Unspecified | Negative |
| Case1006 | ARVC | PanCardiomyopathy | Female | 30 yrs | White | Negative |
| Case1007 | HCM | HCM | Female | 17 yrs | White | Negative |
| Case1008 | HCM | HCM | Male | 37 yrs | White | Negative |
| Case1009 | HCM | HCM | Female | 48 yrs | White | Inconclusive |
| Case1010 | DCM | PanCardiomyopathy | Female | 66 yrs | White | Positive |
| Case1011 | Cardiomyopathy NOS | PanCardiomyopathy | Female | 20 yrs | White | Inconclusive |
| Case1012 | DCM | PanCardiomyopathy | Male | 23 yrs | White | Inconclusive |
| Case1013 | HCM | PanCardiomyopathy | Female | 35 yrs | Asian | Inconclusive |
| Case1014 | HCM | PanCardiomyopathy | Female | 53 yrs | White | Inconclusive |
| Case1015 | DCM | DCM | Male | 44 yrs | Black or African American | Inconclusive |
| Case1016 | DCM | PanCardiomyopathy | Female | 1 yrs | White | Inconclusive |
| Case1017 | HCM | PanCardiomyopathy | Male | 1 yrs | White | Negative |
| Case1018 | ARVC | ARVC | Female | 35 yrs | White | Positive |
| Case1019 | HCM | PanCardiomyopathy | Female | 27 yrs | White | Negative |
| Case1020 | LVNC | LVNC | Male | 40 yrs | White | Inconclusive |
| Case1021 | DCM | DCM | Male | 53 yrs | Mixed | Inconclusive |
| Case1022 | HCM | HCM | Male | 45 yrs | White | Inconclusive |
| Case1023 | ARVC | ARVC | Male | 69 yrs | Unspecified | Negative |
| Case1024 | DCM | PanCardiomyopathy | Female | 2 mos | White | Negative |
| Case1025 | HCM | HCM | Female | 85 yrs | Unspecified | Negative |
| Case1026 | HCM | HCM | Female | 45 yrs | Asian | Inconclusive |
| Case1027 | ARVC | PanCardiomyopathy | Male | 70 yrs | White | Inconclusive |
| Case1028 | LVNC | PanCardiomyopathy | Male | 15 yrs | White | Inconclusive |
| Case1029 | HCM | PanCardiomyopathy | Male | 3 yrs | Black or African American | Inconclusive |
| Case1030 | RCM | PanCardiomyopathy | Female | 1 yrs | Mixed | Positive |
| Case1031 | DCM | PanCardiomyopathy | Male | 50 yrs | White | Inconclusive |
| Case1032 | ARVC | PanCardiomyopathy | Male | 38 yrs | White | Inconclusive |
| Case1033 | DCM | PanCardiomyopathy | Female | 42 yrs | Unspecified | Inconclusive |
| Case1034 | DCM | DCM | Female | 14 yrs | Unspecified | Inconclusive |
| Case1035 | DCM | DCM | Male | 7 yrs | White | Inconclusive |
| Case1036 | DCM | DCM | Male | 53 yrs | Unspecified | Inconclusive |
| Case1037 | HCM | HCM | Male | 17 yrs | White | Inconclusive |
| Case1038 | ARVC | ARVC | Male | 70 yrs | White | Negative |
| Case1039 | HCM | HCM | Male | 57 yrs | White | Negative |
| Case1040 | HCM | HCM | Male | 63 yrs | White | Negative |
| Case1041 | DCM, LVNC | PanCardiomyopathy | Female | 2 mos | Unspecified | Negative |
| Case1042 | DCM | PanCardiomyopathy | Female | 15 yrs | Unspecified | Inconclusive |
| Case1043 | HCM | PanCardiomyopathy | Male | 63 yrs | White | Inconclusive |
| Case1044 | HCM | PanCardiomyopathy | Female | 1 mos | Hispanic or Latino | Inconclusive |
| Case1045 | DCM | PanCardiomyopathy | Female | 7 mos | Hispanic or Latino | Inconclusive |
| Case1046 | DCM | DCM | Female | 9 mos | Unspecified | Inconclusive |
| Case1047 | HCM | PanCardiomyopathy | Male | 1 yrs | White | Inconclusive |
| Case1048 | DCM | PanCardiomyopathy | Male | 12 yrs | Unspecified | Positive |
| Case1049 | HCM | HCM | Male |  | Unspecified | Negative |
| Case1050 | HCM | PanCardiomyopathy | Female | 36 yrs | Unspecified | Positive |
| Case1051 | HCM | PanCardiomyopathy | Female | 60 yrs | White | Inconclusive |
| Case1052 | DCM | PanCardiomyopathy | Male | 42 yrs | White | Negative |
| Case1053 | DCM | PanCardiomyopathy | Male | 68 yrs | Black or African American | Negative |
| Case1054 | DCM | PanCardiomyopathy | Female | 77 yrs | White | Inconclusive |
| Case1055 | ARVC | PanCardiomyopathy | Female | 31 yrs | Unspecified | Positive |
| Case1056 | DCM | PanCardiomyopathy | Male | 3 mos | Black or African American | Positive |
| Case1057 | HCM | HCM | Male | 49 yrs | White | Negative |
| Case1058 | HCM | HCM | Female | 62 yrs | Unspecified | Positive |
| Case1059 | RCM | PanCardiomyopathy | Male | 10 yrs | White | Positive |
| Case1060 | DCM | PanCardiomyopathy | Female | 39 yrs | Black or African American | Positive |
| Case1061 | HCM | HCM | Male | 12 yrs | Unspecified | Positive |
| Case1062 | HCM | PanCardiomyopathy | Male | 9 mos | Unspecified | Inconclusive |
| Case1063 | HCM | PanCardiomyopathy | Female | 62 yrs | Unspecified | Inconclusive |
| Case1064 | LVNC | LVNC | Male | 31 yrs | White | Negative |
| Case1065 | HCM | HCM | Female | 70 yrs | White | Inconclusive |
| Case1066 | ARVC | PanCardiomyopathy | Female | 33 yrs | White | Inconclusive |
| Case1067 | DCM | PanCardiomyopathy | Female |  | White | Negative |
| Case1068 | DCM | PanCardiomyopathy | Female | 3 mos | White | Inconclusive |
| Case1069 | LVNC | PanCardiomyopathy | Male | 3 mos | Black or African American | Inconclusive |
| Case1070 | DCM | PanCardiomyopathy | Female | 5 yrs | Black or African American | Positive |
| Case1071 | DCM | DCM | Female | 12 yrs | Hispanic or Latino | Positive |
| Case1072 | LVNC | PanCardiomyopathy | Female | 15 yrs | White | Negative |
| Case1073 | HCM | HCM | Male | 44 yrs | White | Inconclusive |
| Case1074 | RCM | PanCardiomyopathy | Female | 2 yrs | White | Inconclusive |
| Case1075 | DCM | PanCardiomyopathy | Female | 55 yrs | White | Negative |
| Case1076 | HCM | HCM | Male | 63 yrs | White | Negative |
| Case1077 | HCM | PanCardiomyopathy | Male | 71 yrs | Asian | Inconclusive |
| Case1078 | HCM | HCM | Male | 58 yrs | Unspecified | Inconclusive |
| Case1079 | HCM | HCM | Female | 45 yrs | White | Negative |
| Case1080 | HCM | HCM | Male | 40 yrs | Unspecified | Positive |
| Case1081 | HCM | PanCardiomyopathy | Female | 53 yrs | Unspecified | Negative |
| Case1082 | HCM | HCM | Female | 46 yrs | White | Negative |
| Case1083 | DCM | DCM | Female | 55 yrs | Unspecified | Inconclusive |
| Case1084 | HCM | HCM | Male | 55 yrs | White | Negative |
| Case1085 | RCM | PanCardiomyopathy | Female | 18 yrs | White | Inconclusive |
| Case1086 | HCM | PanCardiomyopathy | Female | 63 yrs | Asian | Inconclusive |
| Case1087 | ARVC | PanCardiomyopathy | Female | 78 yrs | White | Inconclusive |
| Case1088 | DCM | PanCardiomyopathy | Female | 10 yrs | Black or African American | Inconclusive |
| Case1089 | DCM | DCM | Male | 13 yrs | Black or African American | Positive |
| Case1090 | DCM | DCM | Male | 66 yrs | White | Inconclusive |
| Case1091 | DCM | PanCardiomyopathy | Male | 1 mos | Hispanic or Latino | Inconclusive |
| Case1092 | DCM | PanCardiomyopathy | Female | 11 yrs | Black or African American | Inconclusive |
| Case1093 | HCM | HCM | Female | 77 yrs | White | Negative |
| Case1094 | DCM | DCM | Male | 43 yrs | White | Positive |
| Case1095 | HCM | HCM | Male | 33 yrs | Asian | Negative |
| Case1096 | DCM | PanCardiomyopathy | Female | 2 mos | Black or African American | Inconclusive |
| Case1097 | HCM | PanCardiomyopathy | Female | 58 yrs | White | Inconclusive |
| Case1098 | HCM | HCM | Male | 16 yrs | White | Negative |
| Case1099 | HCM | PanCardiomyopathy | Female | 16 yrs | Unspecified | Inconclusive |
| Case1100 | HCM | HCM | Female | 52 yrs | White | Inconclusive |
| Case1101 | DCM | DCM | Male | 46 yrs | White | Negative |
| Case1102 | DCM | DCM | Male | 65 yrs | White | Inconclusive |
| Case1103 | DCM | PanCardiomyopathy | Male | 41 yrs | White | Inconclusive |
| Case1104 | HCM | HCM | Male | 49 yrs | Mixed | Inconclusive |
| Case1105 | HCM | PanCardiomyopathy | Male | 24 yrs | White | Positive |
| Case1106 | DCM | PanCardiomyopathy | Female | 66 yrs | White | Inconclusive |
| Case1107 | HCM | PanCardiomyopathy | Male | 21 yrs | White | Negative |
| Case1108 | Cardiomyopathy NOS | PanCardiomyopathy | Female |  | White | Inconclusive |
| Case1109 | DCM | DCM | Female | 29 yrs | White | Positive |
| Case1110 | ARVC | PanCardiomyopathy | Male | 43 yrs | Unspecified | Inconclusive |
| Case1111 | DCM | PanCardiomyopathy | Male | 4 mos | White | Inconclusive |
| Case1112 | HCM | HCM | Female |  | White | Negative |
| Case1113 | DCM | PanCardiomyopathy | Female | 70 yrs | White | Positive |
| Case1114 | RCM | PanCardiomyopathy | Male | 17 yrs | White | Negative |
| Case1115 | HCM | HCM | Male | 15 yrs | White | Negative |
| Case1116 | HCM | HCM | Male | 24 yrs | Hispanic or Latino | Positive |
| Case1117 | Cardiomyopathy NOS | PanCardiomyopathy | Male | 10 yrs | Unspecified | Inconclusive |
| Case1118 | DCM | DCM | Male |  | White | Inconclusive |
| Case1119 | HCM | PanCardiomyopathy | Male | 16 yrs | American Indian or Alaska Native | Inconclusive |
| Case1120 | DCM | PanCardiomyopathy | Male | 31 yrs | Unspecified | Inconclusive |
| Case1121 | HCM | PanCardiomyopathy | Male | 20 yrs | Hispanic or Latino | Inconclusive |
| Case1122 | Cardiomyopathy NOS | HCM | Female | 62 yrs | Unspecified | Negative |
| Case1123 | DCM | DCM | Male | 20 yrs | White | Positive |
| Case1124 | HCM | PanCardiomyopathy | Unspecified | 37 yrs | White | Inconclusive |
| Case1125 | DCM | PanCardiomyopathy |  | 29 yrs | White | Positive |
| Case1126 | DCM | PanCardiomyopathy | Female | 6 yrs | Unspecified | Inconclusive |
| Case1127 | DCM | PanCardiomyopathy | Male | 40 yrs | White | Inconclusive |
| Case1128 | ARVC | ARVC | Male | 45 yrs | Unspecified | Negative |
| Case1129 | DCM | PanCardiomyopathy | Female | 65 yrs | Unspecified | Inconclusive |
| Case1130 | DCM | PanCardiomyopathy | Male | 52 yrs | Ashkenazi Jewish | Inconclusive |
| Case1131 | DCM | PanCardiomyopathy | Male | 10 yrs | White | Inconclusive |
| Case1132 | DCM | PanCardiomyopathy | Female | 28 yrs | White | Negative |
| Case1133 | DCM | DCM | Male | 41 yrs | White | Negative |
| Case1134 | DCM | PanCardiomyopathy | Male | 15 yrs | White | Positive |
| Case1135 | HCM | PanCardiomyopathy | Male | 40 yrs | Unspecified | Positive |
| Case1136 | LVNC | LVNC | Male | 2 mos | Asian | Negative |
| Case1137 | HCM | PanCardiomyopathy | Female | 57 yrs | Black or African American | Inconclusive |
| Case1138 | DCM | PanCardiomyopathy | Female | 1 mos | Hispanic or Latino | Negative |
| Case1139 | HCM | HCM | Male | 49 yrs | White | Negative |
| Case1140 | HCM | HCM | Male | 10 yrs | Hispanic or Latino | Positive |
| Case1141 | LVNC | PanCardiomyopathy | Male | 47 yrs | White | Inconclusive |
| Case1142 | HCM | PanCardiomyopathy | Male | 10 yrs | Unspecified | Positive |
| Case1143 | HCM | HCM | Male | 68 yrs | Unspecified | Negative |
| Case1144 | DCM | PanCardiomyopathy | Male | 9 yrs | Unspecified | Inconclusive |
| Case1145 | HCM | HCM | Male | 60 yrs | Unspecified | Negative |
| Case1146 | HCM | PanCardiomyopathy | Male | 17 yrs | Unspecified | Inconclusive |
| Case1147 | HCM | PanCardiomyopathy | Female | 36 yrs | Unspecified | Positive |
| Case1148 | RCM | PanCardiomyopathy | Female | 5 yrs | Unspecified | Inconclusive |
| Case1149 | DCM | PanCardiomyopathy | Unspecified | 2 yrs | White | Inconclusive |
| Case1150 | DCM | DCM | Male | 22 yrs | White | Positive |
| Case1151 | Cardiomyopathy NOS | PanCardiomyopathy | Male | 1 yrs | Unspecified | Inconclusive |
| Case1152 | DCM | PanCardiomyopathy | Male | 11 yrs | Ashkenazi Jewish | Negative |
| Case1153 | DCM | PanCardiomyopathy | Male | 15 yrs | White | Inconclusive |
| Case1154 | HCM | HCM | Female | 41 yrs | Unspecified | Negative |
| Case1155 | DCM | PanCardiomyopathy | Female | 16 yrs | Asian | Inconclusive |
| Case1156 | HCM, RCM | PanCardiomyopathy | Female | 2 mos | Asian | Positive |
| Case1157 | Cardiomyopathy NOS | PanCardiomyopathy | Female | 1 mos | White | Negative |
| Case1158 | HCM | HCM | Female | 57 yrs | Black or African American | Inconclusive |
| Case1159 | HCM | PanCardiomyopathy | Male | 43 yrs | Unspecified | Positive |
| Case1160 | DCM | PanCardiomyopathy | Male | 56 yrs | White | Negative |
| Case1161 | HCM | PanCardiomyopathy | Male | 27 yrs | Ashkenazi Jewish | Positive |
| Case1162 | HCM | HCM | Male | 3 yrs | White | Positive |
| Case1163 | DCM | DCM | Male | 48 yrs | Unspecified | Negative |
| Case1164 | DCM | DCM | Female | 67 yrs | Unspecified | Inconclusive |
| Case1165 | HCM | HCM | Female | 73 yrs | White | Inconclusive |
| Case1166 | DCM | PanCardiomyopathy | Male | 1 mos | Hispanic or Latino | Inconclusive |
| Case1167 | HCM | HCM | Female | 59 yrs | White | Positive |
| Case1168 | DCM | PanCardiomyopathy | Male | 1 mos | White | Inconclusive |
| Case1169 | LVNC | LVNC | Female | 34 yrs | White | Inconclusive |
| Case1170 | HCM | HCM | Male | 68 yrs | White | Inconclusive |
| Case1171 | DCM | PanCardiomyopathy | Female | 26 yrs | White | Negative |
| Case1172 | DCM | PanCardiomyopathy | Female | 50 yrs | White | Negative |
| Case1173 | DCM | PanCardiomyopathy | Male | 13 yrs | Unspecified | Inconclusive |
| Case1174 | HCM | HCM | Female | 51 yrs | Unspecified | Positive |
| Case1175 | DCM | DCM | Female | 46 yrs | Unspecified | Inconclusive |
| Case1176 | HCM | HCM | Male | 26 yrs | White | Positive |
| Case1177 | DCM | PanCardiomyopathy | Male | 34 yrs | Mixed | Negative |
| Case1178 | DCM | PanCardiomyopathy | Male | 40 yrs | Mixed | Negative |
| Case1179 | HCM | PanCardiomyopathy | Male | 57 yrs | White | Negative |
| Case1180 | HCM | PanCardiomyopathy | Male | 62 yrs | White | Positive |
| Case1181 | DCM | PanCardiomyopathy | Female | 53 yrs | White | Inconclusive |
| Case1182 | HCM | PanCardiomyopathy | Female |  | White | Inconclusive |
| Case1183 | DCM | PanCardiomyopathy | Female | 7 yrs | White | Inconclusive |
| Case1184 | DCM | PanCardiomyopathy | Male | 11 yrs | White | Inconclusive |
| Case1185 | DCM | PanCardiomyopathy | Female | 16 yrs | Unspecified | Negative |
| Case1186 | HCM | HCM | Female | 20 yrs | Black or African American | Negative |
| Case1187 | HCM | PanCardiomyopathy | Male | 23 yrs | Unspecified | Inconclusive |
| Case1188 | HCM | HCM | Male | 28 yrs | Black or African American | Negative |
| Case1189 | DCM | PanCardiomyopathy | Female | 55 yrs | Black or African American | Inconclusive |
| Case1190 | DCM | DCM | Male | 14 yrs | White | Inconclusive |
| Case1191 | HCM | PanCardiomyopathy | Male | 9 mos | White | Inconclusive |
| Case1192 | HCM | HCM | Female | 2 mos | Asian | Negative |
| Case1193 | DCM | PanCardiomyopathy | Male | 10 mos | White | Inconclusive |
| Case1194 | DCM | PanCardiomyopathy | Male | 1 yrs | White | Inconclusive |
| Case1195 | HCM | HCM | Male | 53 yrs | White | Inconclusive |
| Case1196 | DCM | PanCardiomyopathy | Male | 4 mos | White | Positive |
| Case1197 | HCM | HCM | Male | 61 yrs | White | Negative |
| Case1198 | DCM | PanCardiomyopathy | Male | 19 yrs | White | Inconclusive |
| Case1199 | HCM | PanCardiomyopathy | Female | 37 yrs | White | Positive |
| Case1200 | HCM | PanCardiomyopathy | Male | 56 yrs | Asian | Positive |
| Case1201 | HCM | HCM | Male | 61 yrs | White | Negative |
| Case1202 | HCM | PanCardiomyopathy | Female | 6 mos | Black or African American | Inconclusive |
| Case1203 | HCM | PanCardiomyopathy | Male | 60 yrs | Asian | Positive |
| Case1204 | LVNC | LVNC | Male | 40 yrs | White | Positive |
| Case1205 | HCM | HCM | Male | 58 yrs | Unspecified | Inconclusive |
| Case1206 | HCM | PanCardiomyopathy | Female | 14 yrs | White | Inconclusive |
| Case1207 | DCM | PanCardiomyopathy | Female | 49 yrs | Black or African American | Inconclusive |
| Case1208 | HCM | PanCardiomyopathy | Male | 17 yrs | Unspecified | Inconclusive |
| Case1209 | DCM | PanCardiomyopathy | Male | 21 yrs | White | Inconclusive |
| Case1210 | DCM | PanCardiomyopathy | Male | 3 mos | White | Inconclusive |
| Case1211 | ARVC, ARVC, RCM | PanCardiomyopathy | Male | 65 yrs | Unspecified | Inconclusive |
| Case1212 | DCM | PanCardiomyopathy | Male | 4 mos | White | Negative |
| Case1213 | HCM | HCM | Male | 31 yrs | Unspecified | Inconclusive |
| Case1214 | HCM | HCM | Male | 10 mos | Unspecified | Inconclusive |
| Case1215 | HCM | HCM | Male | 84 yrs | White | Negative |
| Case1216 | HCM | HCM | Male | 26 yrs | Unspecified | Inconclusive |
| Case1217 | HCM | PanCardiomyopathy | Male | 64 yrs | Asian | Inconclusive |
| Case1218 | HCM | PanCardiomyopathy | Male |  | White | Positive |
| Case1219 | HCM | PanCardiomyopathy | Female | 60 yrs | White | Inconclusive |
| Case1220 | ARVC | PanCardiomyopathy | Unspecified | 45 yrs | Unspecified | Positive |
| Case1221 | HCM | HCM | Male | 78 yrs | White | Negative |
| Case1222 | HCM | PanCardiomyopathy | Male | 49 yrs | Asian | Positive |
| Case1223 | HCM | PanCardiomyopathy | Male | 39 yrs | White | Negative |
| Case1224 | ARVC | PanCardiomyopathy | Male | 63 yrs | White | Inconclusive |
| Case1225 | DCM | PanCardiomyopathy | Female | 32 yrs | Unspecified | Inconclusive |
| Case1226 | HCM | PanCardiomyopathy | Male | 11 yrs | White | Positive |
| Case1227 | DCM | DCM | Female | 80 yrs | Unspecified | Positive |
| Case1228 | DCM | PanCardiomyopathy | Female | 33 yrs | White | Positive |
| Case1229 | HCM | HCM | Male | 33 yrs | White | Inconclusive |
| Case1230 | DCM | DCM | Male | 25 yrs | White | Negative |
| Case1231 | DCM | DCM | Male | 25 yrs | White | Inconclusive |
| Case1232 | DCM | PanCardiomyopathy | Female | 65 yrs | Asian | Inconclusive |
| Case1233 | ARVC | PanCardiomyopathy | Male | 15 yrs | Unspecified | Positive |
| Case1234 | Cardiomyopathy NOS | PanCardiomyopathy | Male | 73 yrs | Unspecified | Inconclusive |
| Case1235 | DCM | PanCardiomyopathy | Male | 43 yrs | White | Negative |
| Case1236 | DCM | PanCardiomyopathy | Female | 1 yrs | Unspecified | Inconclusive |
| Case1237 | DCM | PanCardiomyopathy | Male | 1 mos | White | Negative |
| Case1238 | HCM | HCM | Male | 21 yrs | Asian | Inconclusive |
| Case1239 | HCM | HCM | Female | 63 yrs | White | Negative |
| Case1240 | HCM | HCM | Male | 70 yrs | Unspecified | Positive |
| Case1241 | DCM | PanCardiomyopathy | Male | 61 yrs | Ashkenazi Jewish | Inconclusive |
| Case1242 | DCM | PanCardiomyopathy | Male | 35 yrs | White | Inconclusive |
| Case1243 | DCM | PanCardiomyopathy | Male | 52 yrs | White | Inconclusive |
| Case1244 | ARVC | PanCardiomyopathy | Male | 51 yrs | White | Inconclusive |
| Case1245 | HCM | HCM | Female | 76 yrs | Asian | Positive |
| Case1246 | HCM | PanCardiomyopathy | Male | 13 yrs | White | Inconclusive |
| Case1247 | HCM | HCM | Male | 48 yrs | White | Inconclusive |
| Case1248 | DCM | PanCardiomyopathy | Male | 34 yrs | White | Positive |
| Case1249 | HCM | HCM | Male | 33 yrs | Unspecified | Negative |
| Case1250 | HCM | HCM | Female | 70 yrs | White | Positive |
| Case1251 | DCM | PanCardiomyopathy | Male | 11 yrs | White | Negative |
| Case1252 | HCM | PanCardiomyopathy | Male | 42 yrs | Unspecified | Positive |
| Case1253 | HCM | HCM | Male | 61 yrs | Asian | Inconclusive |
| Case1254 | DCM | PanCardiomyopathy | Female | 56 yrs | White | Inconclusive |
| Case1255 | HCM | PanCardiomyopathy | Female | 53 yrs | White | Inconclusive |
| Case1256 | DCM | PanCardiomyopathy | Female | 56 yrs | White | Positive |
| Case1257 | RCM | PanCardiomyopathy | Male | 16 yrs | Unspecified | Inconclusive |
| Case1258 | HCM | HCM | Female | 68 yrs | Unspecified | Negative |
| Case1259 | HCM | PanCardiomyopathy | Male | 61 yrs | Asian | Inconclusive |
| Case1260 | DCM | PanCardiomyopathy | Female | 68 yrs | Black or African American | Inconclusive |
| Case1261 | HCM | PanCardiomyopathy | Male | 1 yrs | White | Positive |
| Case1262 | DCM | PanCardiomyopathy | Male | 54 yrs | White | Inconclusive |
| Case1263 | DCM | PanCardiomyopathy | Male | 21 yrs | White | Positive |
| Case1264 | HCM | HCM | Female | 38 yrs | White | Negative |
| Case1265 | HCM | HCM | Female | 55 yrs | White | Negative |
| Case1266 | HCM | PanCardiomyopathy | Female | 57 yrs | White | Inconclusive |
| Case1267 | DCM | DCM | Male | 22 yrs | Black or African American | Inconclusive |
| Case1268 | DCM | PanCardiomyopathy | Female | 9 mos | Unspecified | Inconclusive |
| Case1269 | HCM | HCM | Female | 54 yrs | White | Negative |
| Case1270 | HCM | PanCardiomyopathy | Female | 55 yrs | White | Negative |
| Case1271 | HCM | PanCardiomyopathy | Male | 44 yrs | Ashkenazi Jewish | Inconclusive |
| Case1272 | DCM | PanCardiomyopathy | Male | 13 yrs | White | Inconclusive |
| Case1273 | HCM | HCM | Male | 37 yrs | Unspecified | Positive |
| Case1274 | DCM | PanCardiomyopathy | Male | 25 yrs | White | Negative |
| Case1275 | HCM | PanCardiomyopathy | Male | 19 yrs | White | Inconclusive |
| Case1276 | DCM | PanCardiomyopathy | Male |  | Unspecified | Negative |
| Case1277 | HCM | HCM | Male | 57 yrs | Unspecified | Inconclusive |
| Case1278 | DCM | PanCardiomyopathy | Male | 16 yrs | Black or African American | Inconclusive |
| Case1279 | Cardiomyopathy NOS | PanCardiomyopathy | Female | 1 yrs | White | Inconclusive |
| Case1280 | HCM | HCM | Male | 13 yrs | Unspecified | Positive |
| Case1281 | DCM | PanCardiomyopathy | Male | 29 yrs | White | Negative |
| Case1282 | DCM | PanCardiomyopathy | Female | 41 yrs | White | Inconclusive |
| Case1283 | DCM | PanCardiomyopathy | Female | 13 yrs | Hispanic or Latino | Inconclusive |
| Case1284 | HCM | PanCardiomyopathy | Female |  | White | Inconclusive |
| Case1285 | DCM | PanCardiomyopathy | Male | 2 mos | Unspecified | Inconclusive |
| Case1286 | HCM | PanCardiomyopathy | Female | 17 yrs | Mixed | Positive |
| Case1287 | ARVC | ARVC | Male | 16 yrs | White | Positive |
| Case1288 | HCM | HCM | Male | 40 yrs | Unspecified | Negative |
| Case1289 | DCM | DCM | Male | 50 yrs | White | Inconclusive |
| Case1290 | DCM | PanCardiomyopathy | Male | 65 yrs | White | Positive |
| Case1291 | HCM | PanCardiomyopathy | Male | 61 yrs | Ashkenazi Jewish | Inconclusive |
| Case1292 | HCM | HCM | Female | 17 yrs | Hispanic or Latino | Positive |
| Case1293 | HCM | HCM | Female | 69 yrs | White | Negative |
| Case1294 | HCM | HCM | Female | 64 yrs | White | Negative |
| Case1295 | DCM | PanCardiomyopathy | Male | 7 mos | Hispanic or Latino | Inconclusive |
| Case1296 | HCM | PanCardiomyopathy | Male | 66 yrs | White | Inconclusive |
| Case1297 | HCM | PanCardiomyopathy | Female | 76 yrs | Asian | Inconclusive |
| Case1298 | HCM | HCM | Female | 66 yrs | White | Inconclusive |
| Case1299 | DCM | PanCardiomyopathy | Male |  | White | Inconclusive |
| Case1300 | DCM | PanCardiomyopathy | Male | 70 yrs | Unspecified | Inconclusive |
| Case1301 | DCM | PanCardiomyopathy | Female | 48 yrs | White | Positive |
| Case1302 | DCM | PanCardiomyopathy | Male | 1 mos | Hispanic or Latino | Positive |
| Case1303 | DCM | PanCardiomyopathy | Female | 37 yrs | Unspecified | Negative |
| Case1304 | Cardiomyopathy NOS | PanCardiomyopathy | Male | 1 mos | Unspecified | Inconclusive |
| Case1305 | DCM | PanCardiomyopathy | Female | 50 yrs | Black or African American | Inconclusive |
| Case1306 | HCM | PanCardiomyopathy | Male | 66 yrs | White | Inconclusive |
| Case1307 | HCM | HCM | Male | 59 yrs | White | Negative |
| Case1308 | DCM | DCM | Unspecified | 23 yrs | White | Inconclusive |
| Case1309 | LVNC | LVNC | Male | 25 yrs | White | Negative |
| Case1310 | HCM | HCM | Male | 52 yrs | White | Negative |
| Case1311 | LVNC | PanCardiomyopathy | Male | 36 yrs | Mixed | Inconclusive |
| Case1312 | HCM | HCM | Female | 80 yrs | Hispanic or Latino | Inconclusive |
| Case1313 | DCM | PanCardiomyopathy | Female | 16 yrs | White | Inconclusive |
| Case1314 | HCM | HCM | Male | 48 yrs | White | Negative |
| Case1315 | DCM | DCM | Female | 47 yrs | White | Negative |
| Case1316 | HCM | HCM | Male | 73 yrs | Unspecified | Negative |
| Case1317 | LVNC | LVNC | Male | 9 mos | White | Negative |
| Case1318 | HCM | HCM | Female | 89 yrs | White | Negative |
| Case1319 | HCM | HCM | Male | 55 yrs | White | Negative |
| Case1320 | HCM | HCM | Male | 49 yrs | White | Negative |
| Case1321 | HCM | HCM | Male | 62 yrs | White | Inconclusive |
| Case1322 | DCM | PanCardiomyopathy | Male | 3 mos | Hispanic or Latino | Inconclusive |
| Case1323 | HCM | HCM | Male | 53 yrs | Unspecified | Negative |
| Case1324 | HCM | PanCardiomyopathy | Female | 60 yrs | Asian | Inconclusive |
| Case1325 | HCM | HCM | Male | 60 yrs | White | Negative |
| Case1326 | DCM | PanCardiomyopathy | Female | 1 yrs | Unspecified | Inconclusive |
| Case1327 | LVNC | PanCardiomyopathy | Male | 14 yrs | Unspecified | Inconclusive |
| Case1328 | HCM | PanCardiomyopathy | Male | 19 yrs | White | Inconclusive |
| Case1329 | HCM | HCM | Male | 61 yrs | Unspecified | Inconclusive |
| Case1330 | HCM | HCM | Male | 54 yrs | White | Positive |
| Case1331 | HCM | HCM | Female | 62 yrs | White | Negative |
| Case1332 | DCM | PanCardiomyopathy | Male | 14 yrs | White | Inconclusive |
| Case1333 | Cardiomyopathy NOS | PanCardiomyopathy | Male | 19 yrs | White | Inconclusive |
| Case1334 | HCM | PanCardiomyopathy | Female | 46 yrs | Black or African American | Inconclusive |
| Case1335 | DCM | PanCardiomyopathy | Male | 30 yrs | White | Inconclusive |
| Case1336 | HCM | PanCardiomyopathy | Male | 63 yrs | White | Negative |
| Case1337 | HCM | PanCardiomyopathy | Male | 65 yrs | Asian | Negative |
| Case1338 | ARVC | ARVC | Male | 52 yrs | White | Negative |
| Case1339 | HCM | HCM | Male | 57 yrs | Unspecified | Negative |
| Case1340 | DCM | PanCardiomyopathy | Male | 15 yrs | Mixed | Negative |
| Case1341 | HCM | HCM | Male | 70 yrs | White | Negative |
| Case1342 | DCM | PanCardiomyopathy | Female | 70 yrs | White | Inconclusive |
| Case1343 | ARVC | PanCardiomyopathy | Male | 39 yrs | White | Inconclusive |
| Case1344 | HCM | HCM | Unspecified | 58 yrs | White | Negative |
| Case1345 | HCM | HCM | Male | 47 yrs | White | Negative |
| Case1346 | HCM | HCM | Female | 82 yrs | White | Negative |
| Case1347 | DCM | PanCardiomyopathy | Male | 54 yrs | White | Inconclusive |
| Case1348 | HCM | HCM | Male | 17 yrs | Unspecified | Negative |
| Case1349 | DCM | DCM | Female | 47 yrs | Unspecified | Inconclusive |
| Case1350 | ARVC | ARVC | Male | 36 yrs | Native Hawaiian or Other Pacific Islander | Negative |
| Case1351 | HCM | PanCardiomyopathy | Male | 1 yrs | Black or African American | Inconclusive |
| Case1352 | HCM | PanCardiomyopathy | Female | 61 yrs | Ashkenazi Jewish | Inconclusive |
| Case1353 | HCM | PanCardiomyopathy | Male | 52 yrs | White | Inconclusive |
| Case1354 | HCM | HCM | Male | 58 yrs | White | Negative |
| Case1355 | HCM | HCM | Male | 37 yrs | White | Positive |
| Case1356 | DCM | PanCardiomyopathy | Female | 22 yrs | White | Positive |
| Case1357 | HCM | HCM | Male | 56 yrs | White | Inconclusive |
| Case1358 | DCM | PanCardiomyopathy | Male | 16 yrs | Mixed | Inconclusive |
| Case1359 | HCM | HCM | Female | 68 yrs | White | Inconclusive |
| Case1360 | DCM | PanCardiomyopathy | Female | 4 mos | Black or African American | Inconclusive |
| Case1361 | HCM | HCM | Female | 22 yrs | Unspecified | Negative |
| Case1362 | HCM | HCM | Male | 16 yrs | Mixed | Negative |
| Case1363 | DCM | PanCardiomyopathy | Male | 3 mos | White | Inconclusive |
| Case1364 | HCM | PanCardiomyopathy | Male | 52 yrs | White | Inconclusive |
| Case1365 | HCM | HCM | Male | 49 yrs | Unspecified | Negative |
| Case1366 | DCM | PanCardiomyopathy | Female | 8 yrs | Unspecified | Inconclusive |
| Case1367 | HCM | HCM | Male | 61 yrs | White | Negative |
| Case1368 | DCM | PanCardiomyopathy | Male | 52 yrs | White | Inconclusive |
| Case1369 | HCM | PanCardiomyopathy | Male | 66 yrs | Unspecified | Positive |
| Case1370 | ARVC | PanCardiomyopathy | Female | 55 yrs | White | Inconclusive |
| Case1371 | DCM | PanCardiomyopathy | Male | 48 yrs | Unspecified | Inconclusive |
| Case1372 | DCM | PanCardiomyopathy | Male | 47 yrs | White | Inconclusive |
| Case1373 | HCM | HCM | Male | 15 yrs | Asian | Negative |
| Case1374 | DCM | PanCardiomyopathy | Male | 1 yrs | Unspecified | Inconclusive |
| Case1375 | DCM | DCM | Female | 64 yrs | White | Inconclusive |
| Case1376 | HCM | HCM | Female | 58 yrs | Asian | Negative |
| Case1377 | HCM | HCM | Male | 65 yrs | White | Negative |
| Case1378 | HCM | HCM | Female | 59 yrs | White | Inconclusive |
| Case1379 | HCM | HCM | Male | 43 yrs | White | Negative |
| Case1380 | HCM | HCM | Female | 62 yrs | White | Inconclusive |
| Case1381 | HCM | HCM | Male | 45 yrs | White | Negative |
| Case1382 | HCM | HCM | Female | 41 yrs | White | Negative |
| Case1383 | DCM | DCM | Male | 22 yrs | White | Negative |
| Case1384 | HCM | HCM | Female | 66 yrs | White | Negative |
| Case1385 | HCM | HCM | Male | 73 yrs | White | Inconclusive |
| Case1386 | HCM | HCM | Male | 61 yrs | White | Negative |
| Case1387 | DCM | PanCardiomyopathy | Female | 1 mos | White | Positive |
| Case1388 | Cardiomyopathy NOS | PanCardiomyopathy | Male | 1 mos | Unspecified | Inconclusive |
| Case1389 | HCM | HCM | Male | 51 yrs | Black or African American | Negative |
| Case1390 | ARVC | ARVC | Male | 38 yrs | Unspecified | Negative |
| Case1391 | HCM | PanCardiomyopathy | Male | 68 yrs | Ashkenazi Jewish | Inconclusive |
| Case1392 | DCM | PanCardiomyopathy | Female | 61 yrs | Mixed | Positive |
| Case1393 | HCM | PanCardiomyopathy | Male | 49 yrs | White | Inconclusive |
| Case1394 | HCM | PanCardiomyopathy | Male | 57 yrs | White | Negative |
| Case1395 | ARVC | PanCardiomyopathy | Male | 53 yrs | Unspecified | Inconclusive |
| Case1396 | DCM | DCM | Male | 63 yrs | White | Inconclusive |
| Case1397 | DCM | DCM | Female | 1 mos | White | Inconclusive |
| Case1398 | HCM | PanCardiomyopathy | Female | 75 yrs | White | Inconclusive |
| Case1399 | HCM | HCM | Male | 23 yrs | Black or African American | Positive |
| Case1400 | DCM | PanCardiomyopathy | Male | 49 yrs | White | Inconclusive |
| Case1401 | HCM | HCM | Male | 35 yrs | White | Positive |
| Case1402 | ARVC | ARVC | Female | 21 yrs | White | Negative |
| Case1403 | HCM | HCM | Male | 64 yrs | White | Positive |
| Case1404 | HCM | PanCardiomyopathy | Female | 46 yrs | Hispanic or Latino | Inconclusive |
| Case1405 | HCM | PanCardiomyopathy | Female | 63 yrs | Unspecified | Positive |
| Case1406 | HCM | PanCardiomyopathy | Female | 58 yrs | White | Negative |
| Case1407 | HCM | PanCardiomyopathy | Male | 34 yrs | White | Positive |
| Case1408 | HCM | HCM | Female | 1 yrs | White | Negative |
| Case1409 | HCM | HCM | Male | 50 yrs | White | Positive |
| Case1410 | HCM | PanCardiomyopathy | Male | 6 yrs | White | Inconclusive |
| Case1411 | DCM | PanCardiomyopathy | Female | 9 mos | White | Inconclusive |
| Case1412 | HCM | HCM | Male | 78 yrs | White | Negative |
| Case1413 | HCM | HCM | Female | 65 yrs | Unspecified | Negative |
| Case1414 | HCM | HCM | Female | 61 yrs | Unspecified | Negative |
| Case1415 | HCM | HCM | Male | 71 yrs | White | Negative |
| Case1416 | HCM | PanCardiomyopathy | Male | 22 yrs | Black or African American | Inconclusive |
| Case1417 | HCM | PanCardiomyopathy | Female | 78 yrs | White | Inconclusive |
| Case1418 | HCM | HCM | Male | 43 yrs | Unspecified | Inconclusive |
| Case1419 | LVNC | PanCardiomyopathy | Female | 34 yrs | Asian | Inconclusive |
| Case1420 | HCM | PanCardiomyopathy | Male | 54 yrs | Asian | Inconclusive |
| Case1421 | DCM | PanCardiomyopathy | Male | 32 yrs | Unspecified | Positive |
| Case1422 | DCM | PanCardiomyopathy | Male | 4 mos | Black or African American | Inconclusive |
| Case1423 | DCM | PanCardiomyopathy | Female | 17 yrs | Black or African American | Inconclusive |
| Case1424 | DCM | PanCardiomyopathy | Male | 4 yrs | Mixed | Inconclusive |
| Case1425 | HCM | PanCardiomyopathy | Male | 39 yrs | White | Positive |

Cardiomyopathy NOS, cardiomyopathy nonspecific; yrs, years; mos, months

**Supplementary Table S3: Frequency of CNVs called by VisCap***

| **interval** | **#exons** | **start** | **end** | **loss freq%** | **gain freq%** |
| --- | --- | --- | --- | --- | --- |
| 10:92678607-92678815 | 1 | ANKRD1_Exon_04 | ANKRD1_Exon_04 | 8.3% | 5.1% |
| 16:30907950-30908004 | 1 | CTF1_Exon_01 | CTF1_Exon_01 | 5.1% | 6.7% |
| 3:14166679-14166720 | 1 | TMEM43_Exon_01 | TMEM43_Exon_01 | 3.1% | 5.2% |
| 7:151329140-151329239 | 1 | PRKAG2_Exon_05 | PRKAG2_Exon_05 | 3.1% | 4.3% |
| 2:179617836-179617922 | 1 | TTN_Exon_45 | TTN_Exon_45 | 3.0% | 0.2% |
| 7:151262417-151262484 | 1 | PRKAG2_Exon_13 | PRKAG2_Exon_13 | 2.9% | 2.1% |
| 1:237527643-237527687 | 1 | RYR2_Exon_05 | RYR2_Exon_05 | 2.9% | 0.4% |
| 10:112404198-112404418 | 1 | RBM20_Exon_01 | RBM20_Exon_01 | 2.7% | 2.3% |
| X:100654735-100654793 | 1 | GLA_Intron_04 | GLA_Intron_04 | 2.7% | 3.0% |
| 15:63340760-63340921 | 1 | TPM1_Exon_02A | TPM1_Exon_02A | 2.6% | 4.3% |
| 1:237881747-237881836 | 1 | RYR2_Exon_73 | RYR2_Exon_73 | 2.6% | 0.0% |
| X:153642423-153642542 | 1 | TAZ_Exon_05 | TAZ_Exon_05 | 2.5% | 3.1% |
| 18:29078200-29078274 | 1 | DSG2_Exon_01 | DSG2_Exon_01 | 2.5% | 3.2% |
| 1:237655091-237655239 | 1 | RYR2_Exon_18 | RYR2_Exon_18 | 2.1% | 0.0% |
| 1:237958574-237958646 | 1 | RYR2_Exon_96 | RYR2_Exon_96 | 1.9% | 0.1% |
| 1:78381777-78381833 | 1 | NEXN_Exon_02 | NEXN_Exon_02 | 1.7% | 2.3% |
| 1:237889558-237889623 | 1 | RYR2_Exon_75 | RYR2_Exon_75 | 1.5% | 0.0% |
| 2:179643941-179644204 | 1 | TTN_Exon_023 | TTN_Exon_023 | 1.5% | 0.0% |
| X:153607830-153607941 | 1 | EMD_Exon_01 | EMD_Exon_01 | 1.5% | 0.8% |
| 1:237831168-237831273 | 1 | RYR2_Exon_58 | RYR2_Exon_58 | 1.4% | 0.4% |
| 10:21148627-21148770 | 1 | NEBL_Exon_08 | NEBL_Exon_08 | 1.4% | 0.0% |
| 2:179505953-179506057 | 1 | TTN_Exon_169 | TTN_Exon_169 | 1.4% | 0.4% |
| X:119590491-119590639 | 1 | LAMP2_Exon_02 | LAMP2_Exon_02 | 1.4% | 1.2% |
| 1:237821229-237821337 | 1 | RYR2_Exon_54 | RYR2_Exon_54 | 1.3% | 0.1% |
| 10:75802826-75802926 | 1 | VCL_Exon_02 | VCL_Exon_02 | 1.3% | 0.2% |
| 12:22017358-22017426 | 1 | ABCC9_Exon_17 | ABCC9_Exon_17 | 1.2% | 0.0% |
| 12:33049428-33049680 | 1 | PKP2_Exon_01 | PKP2_Exon_01 | 1.2% | 1.1% |
| 1:237865263-237865374 | 1 | RYR2_Exon_66 | RYR2_Exon_66 | 1.1% | 0.0% |
| 1:237875030-237875152 | 1 | RYR2_Exon_71 | RYR2_Exon_71 | 1.1% | 0.0% |
| 1:78390858-78390929 | 1 | NEXN_Exon_06 | NEXN_Exon_06 | 1.1% | 0.4% |
| 2:179522789-179522899 | 1 | TTN_Exon_155D | TTN_Exon_155D | 1.1% | 1.1% |
| 2:179654690-179654857 | 1 | TTN_Exon_012 | TTN_Exon_012 | 1.1% | 0.0% |
| X:119602946-119603039 | 1 | LAMP2_Exon_01 | LAMP2_Exon_01 | 1.1% | 1.3% |
| X:153640166-153640343 | 1 | TAZ_Exon_01 | TAZ_Exon_01 | 1.0% | 2.6% |
| 1:237205807-237205884 | 1 | RYR2_Exon_01 | RYR2_Exon_01 | 1.0% | 1.5% |
| 1:237814696-237814816 | 1 | RYR2_Exon_051 | RYR2_Exon_051 | 1.0% | 0.0% |
| 15:63335014-63335157 | 1 | TPM1_Exon_01 | TPM1_Exon_01 | 1.0% | 1.4% |
| 18:29100751-29100942 | 1 | DSG2_Exon_04 | DSG2_Exon_04 | 1.0% | 0.0% |
| 18:29115218-29115390 | 1 | DSG2_Exon_10 | DSG2_Exon_10 | 1.0% | 0.0% |
| 7:151267242-151272050 | 3 | PRKAG2_Exon_10 | PRKAG2_Exon_08 | 1.0% | 0.0% |
| 1:78383630-78383738 | 1 | NEXN_Exon_04 | NEXN_Exon_04 | 0.8% | 0.0% |
| 10:21178759-21178893 | 1 | NEBL_Exon_03 | NEBL_Exon_03 | 0.8% | 0.0% |
| 2:179559541-179559648 | 1 | TTN_Exon_113 | TTN_Exon_113 | 0.8% | 0.0% |
| 1:78390858-78392311 | 2 | NEXN_Exon_06 | NEXN_Exon_07 | 0.8% | 0.0% |
| 2:179547409-179548033 | 2 | TTN_Exon_132 | TTN_Exon_131 | 0.8% | 0.0% |
| 1:156107430-156107570 | 1 | LMNA_Exon_10 | LMNA_Exon_10 | 0.7% | 0.2% |
| 1:237862250-237862340 | 1 | RYR2_Exon_064 | RYR2_Exon_064 | 0.7% | 0.0% |
| 1:237994799-237994880 | 1 | RYR2_Exon_104 | RYR2_Exon_104 | 0.7% | 0.0% |
| 10:21112122-21112244 | 1 | NEBL_Exon_19 | NEBL_Exon_19 | 0.7% | 0.0% |
| 12:21962771-21962904 | 1 | ABCC9_Exon_35 | ABCC9_Exon_35 | 0.7% | 0.0% |
| 2:179539751-179539855 | 1 | TTN_Exon_145 | TTN_Exon_145 | 0.7% | 0.0% |
| 2:220283170-220283777 | 1 | DES_Exon_01 | DES_Exon_01 | 0.7% | 0.2% |
| 2:179505253-179506057 | 2 | TTN_Exon_170 | TTN_Exon_169 | 0.7% | 0.0% |
| 2:179654072-179654857 | 2 | TTN_Exon_013 | TTN_Exon_012 | 0.7% | 0.0% |
| 1:237905586-237905664 | 1 | RYR2_Exon_80 | RYR2_Exon_80 | 0.6% | 0.0% |
| 11:47353407-47353447 | 1 | MYBPC3_Exon_34 | MYBPC3_Exon_34 | 0.6% | 1.2% |
| 11:47371550-47371678 | 1 | MYBPC3_Exon_04 | MYBPC3_Exon_04 | 0.6% | 0.6% |
| 18:32335926-32336022 | 1 | DTNA_Exon_03 | DTNA_Exon_03 | 0.6% | 0.0% |
| 19:55667554-55667715 | 1 | TNNI3_Exon_05 | TNNI3_Exon_05 | 0.6% | 0.7% |
| 2:179394672-179394858 | 1 | TTN_Exon_308 | TTN_Exon_308 | 0.6% | 0.0% |
| 2:179536683-179537009 | 1 | TTN_Exon_150 | TTN_Exon_150 | 0.6% | 0.0% |
| 6:112455654-112455826 | 1 | LAMA4_Exon_26 | LAMA4_Exon_26 | 0.6% | 0.0% |
| 6:7542134-7542333 | 1 | DSP_Exon_01 | DSP_Exon_01 | 0.6% | 0.6% |
| 6:7578682-7578810 | 1 | DSP_Exon_22 | DSP_Exon_22 | 0.6% | 0.0% |
| 1:156084695-156085080 | 1 | LMNA_Exon_01 | LMNA_Exon_01 | 0.5% | 0.1% |
| 1:236849959-236850114 | 1 | ACTN2_Exon_01 | ACTN2_Exon_01 | 0.5% | 0.4% |
| 1:237944850-237944961 | 1 | RYR2_Exon_089 | RYR2_Exon_089 | 0.5% | 0.0% |
| 1:78383236-78383457 | 1 | NEXN_Exon_03 | NEXN_Exon_03 | 0.5% | 0.0% |
| 10:21129653-21129793 | 1 | NEBL_Exon_13 | NEBL_Exon_13 | 0.5% | 0.0% |
| 11:47364796-47364828 | 1 | MYBPC3_Exon_14 | MYBPC3_Exon_14 | 0.5% | 0.6% |
| 11:47368563-47368595 | 1 | MYBPC3_Exon_10 | MYBPC3_Exon_10 | 0.5% | 1.1% |
| 15:63335890-63336045 | 1 | TPM1_Exon_01A | TPM1_Exon_01A | 0.5% | 1.1% |
| 2:179538345-179538452 | 1 | TTN_Exon_147 | TTN_Exon_147 | 0.5% | 0.0% |
| 2:179547923-179548033 | 1 | TTN_Exon_131 | TTN_Exon_131 | 0.5% | 0.0% |
| 2:179569589-179569708 | 1 | TTN_Exon_101 | TTN_Exon_101 | 0.5% | 0.0% |
| 2:179656784-179656939 | 1 | TTN_Exon_010 | TTN_Exon_010 | 0.5% | 0.0% |
| X:153608035-153608169 | 1 | EMD_Exon_02 | EMD_Exon_02 | 0.5% | 0.2% |
| X:153640166-153640304 | 1 | TAZ_Exon_01 | TAZ_Exon_01 | 0.5% | 1.0% |
| 18:28662189-28663041 | 2 | DSC2_Exon_09 | DSC2_Exon_08 | 0.5% | 0.0% |
| 2:179559311-179560152 | 3 | TTN_Exon_114 | TTN_Exon_112 | 0.5% | 0.0% |
| 1:78398952-78399179 | 1 | NEXN_Exon_10 | NEXN_Exon_10 | 0.4% | 0.0% |
| 11:47368163-47368210 | 1 | MYBPC3_Exon_11 | MYBPC3_Exon_11 | 0.4% | 0.7% |
| 11:47369393-47369471 | 1 | MYBPC3_Exon_07 | MYBPC3_Exon_07 | 0.4% | 0.1% |
| 12:21997402-21997501 | 1 | ABCC9_Exon_26 | ABCC9_Exon_26 | 0.4% | 0.0% |
| 12:22063076-22063251 | 1 | ABCC9_Exon_08 | ABCC9_Exon_08 | 0.4% | 0.0% |
| 16:30913384-30913875 | 1 | CTF1_Exon_03 | CTF1_Exon_03 | 0.4% | 0.1% |
| 18:28672049-28672278 | 1 | DSC2_Exon_03 | DSC2_Exon_03 | 0.4% | 0.0% |
| 2:179534930-179535037 | 1 | TTN_Exon_152 | TTN_Exon_152 | 0.4% | 0.0% |
| 2:179560577-179561011 | 1 | TTN_Exon_111 | TTN_Exon_111 | 0.4% | 0.0% |
| 2:179561833-179561910 | 1 | TTN_Exon_110 | TTN_Exon_110 | 0.4% | 0.0% |
| 3:52487993-52488046 | 1 | TNNC1_Exon_01 | TNNC1_Exon_01 | 0.4% | 0.2% |
| 7:151267242-151267326 | 1 | PRKAG2_Exon_10 | PRKAG2_Exon_10 | 0.4% | 0.0% |
| X:153641529-153641604 | 1 | TAZ_Exon_03 | TAZ_Exon_03 | 0.4% | 0.4% |
| X:153648029-153648100 | 1 | TAZ_Exon_07 | TAZ_Exon_07 | 0.4% | 0.0% |
| 1:237519250-237527687 | 2 | RYR2_Exon_004 | RYR2_Exon_005 | 0.4% | 0.0% |
| 1:237821229-237823389 | 2 | RYR2_Exon_054 | RYR2_Exon_055 | 0.4% | 0.0% |
| 1:237935297-237936963 | 2 | RYR2_Exon_086 | RYR2_Exon_087 | 0.4% | 0.0% |
| 10:21148627-21157709 | 2 | NEBL_Exon_08 | NEBL_Exon_07 | 0.4% | 0.0% |
| X:153640166-153640566 | 2 | TAZ_Exon_01 | TAZ_Exon_02 | 0.4% | 0.5% |
| 1:78381777-78383738 | 3 | NEXN_Exon_02 | NEXN_Exon_04 | 0.4% | 0.0% |
| 10:21129653-21139446 | 3 | NEBL_Exon_13 | NEBL_Exon_11 | 0.4% | 0.0% |
| 2:179559311-179561910 | 5 | TTN_Exon_114 | TTN_Exon_110 | 0.4% | 0.0% |
| 1:201328323-201328398 | 1 | TNNT2_Exon_16 | TNNT2_Exon_16 | 0.2% | 0.0% |
| 1:201333411-201333518 | 1 | TNNT2_Exon_10 | TNNT2_Exon_10 | 0.2% | 0.0% |
| 1:237936805-237936963 | 1 | RYR2_Exon_087 | RYR2_Exon_087 | 0.2% | 0.0% |
| 1:78408131-78408529 | 1 | NEXN_Exon_13 | NEXN_Exon_13 | 0.2% | 0.0% |
| 10:92678880-92679040 | 1 | ANKRD1_Exon_03 | ANKRD1_Exon_03 | 0.2% | 0.0% |
| 11:47369187-47369246 | 1 | MYBPC3_Exon_08 | MYBPC3_Exon_08 | 0.2% | 0.2% |
| 12:22028573-22028675 | 1 | ABCC9_Exon_15 | ABCC9_Exon_15 | 0.2% | 0.0% |
| 12:22048194-22048264 | 1 | ABCC9_Exon_11 | ABCC9_Exon_11 | 0.2% | 0.0% |
| 18:28648260-28648325 | 1 | DSC2_Exon_15B | DSC2_Exon_15B | 0.2% | 0.1% |
| 18:28659798-28659970 | 1 | DSC2_Exon_11 | DSC2_Exon_11 | 0.2% | 0.0% |
| 18:28662189-28662404 | 1 | DSC2_Exon_09 | DSC2_Exon_09 | 0.2% | 0.0% |
| 18:29102031-29102227 | 1 | DSG2_Exon_06 | DSG2_Exon_06 | 0.2% | 0.0% |
| 2:179523717-179523830 | 1 | TTN_Exon_155 | TTN_Exon_155 | 0.2% | 0.1% |
| 2:179542333-179542659 | 1 | TTN_Exon_143 | TTN_Exon_143 | 0.2% | 0.0% |
| 2:179546373-179546480 | 1 | TTN_Exon_133 | TTN_Exon_133 | 0.2% | 0.0% |
| 2:179559311-179559418 | 1 | TTN_Exon_114 | TTN_Exon_114 | 0.2% | 0.2% |
| 2:179644712-179644947 | 1 | TTN_Exon_022 | TTN_Exon_022 | 0.2% | 0.0% |
| 7:151271962-151272050 | 1 | PRKAG2_Exon_08 | PRKAG2_Exon_08 | 0.2% | 0.2% |
| X:119562324-119562496 | 1 | LAMP2_Exon_09C | LAMP2_Exon_09C | 0.2% | 0.0% |
| X:153648982-153649089 | 1 | TAZ_Exon_10 | TAZ_Exon_10 | 0.2% | 0.5% |
| 1:78392084-78392592 | 2 | NEXN_Exon_07 | NEXN_Exon_08 | 0.2% | 0.0% |
| 1:78407693-78408529 | 2 | NEXN_Exon_12 | NEXN_Exon_13 | 0.2% | 0.0% |
| 12:22063076-22063927 | 2 | ABCC9_Exon_08 | ABCC9_Exon_07 | 0.2% | 0.0% |
| 2:179549965-179550340 | 2 | TTN_Exon_126 | TTN_Exon_125 | 0.2% | 0.0% |
| 2:179549965-179552966 | 3 | TTN_Exon_126 | TTN_Exon_124 | 0.2% | 0.0% |
| 1:156106889-156107038 | 1 | LMNA_Exon_09 | LMNA_Exon_09 | 0.1% | 0.0% |
| 1:201331499-201331537 | 1 | TNNT2_Exon_12 | TNNT2_Exon_12 | 0.1% | 0.6% |
| 1:201338929-201338988 | 1 | TNNT2_Exon_04A | TNNT2_Exon_04A | 0.1% | 0.0% |
| 1:237765310-237765426 | 1 | RYR2_Exon_035 | RYR2_Exon_035 | 0.1% | 0.0% |
| 1:237920982-237921091 | 1 | RYR2_Exon_082 | RYR2_Exon_082 | 0.1% | 0.0% |
| 1:78383795-78383973 | 1 | NEXN_Exon_05 | NEXN_Exon_05 | 0.1% | 0.0% |
| 1:78392084-78392311 | 1 | NEXN_Exon_07 | NEXN_Exon_07 | 0.1% | 0.0% |
| 10:75757951-75758148 | 1 | VCL_Exon_01 | VCL_Exon_01 | 0.1% | 0.6% |
| 10:88428434-88428556 | 1 | LDB3_Exon_01 | LDB3_Exon_01 | 0.1% | 0.0% |
| 11:47362539-47362598 | 1 | MYBPC3_Exon_20 | MYBPC3_Exon_20 | 0.1% | 0.2% |
| 11:47372038-47372181 | 1 | MYBPC3_Exon_03 | MYBPC3_Exon_03 | 0.1% | 1.3% |
| 12:22013890-22014004 | 1 | ABCC9_Exon_19 | ABCC9_Exon_19 | 0.1% | 0.0% |
| 12:22063745-22063927 | 1 | ABCC9_Exon_07 | ABCC9_Exon_07 | 0.1% | 0.0% |
| 12:22089452-22089623 | 1 | ABCC9_Exon_01 | ABCC9_Exon_01 | 0.1% | 0.0% |
| 17:37821598-37821737 | 1 | TCAP_Exon_01 | TCAP_Exon_01 | 0.1% | 0.0% |
| 17:39920950-39921083 | 1 | JUP_Exon_07 | JUP_Exon_07 | 0.1% | 0.1% |
| 18:29104396-29104563 | 1 | DSG2_Exon_07 | DSG2_Exon_07 | 0.1% | 0.0% |
| 18:32405216-32405254 | 1 | DTNA_Exon_11 | DTNA_Exon_11 | 0.1% | 0.0% |
| 19:55668403-55668517 | 1 | TNNI3_Exon_03 | TNNI3_Exon_03 | 0.1% | 0.2% |
| 19:55668649-55668691 | 1 | TNNI3_Exon_02 | TNNI3_Exon_02 | 0.1% | 0.7% |
| 19:55668921-55668972 | 1 | TNNI3_Exon_01 | TNNI3_Exon_01 | 0.1% | 0.6% |
| 2:179473920-179474312 | 1 | TTN_Exon_222 | TTN_Exon_222 | 0.1% | 0.0% |
| 2:179519157-179519276 | 1 | TTN_Exon_155A | TTN_Exon_155A | 0.1% | 0.0% |
| 2:179531536-179531643 | 1 | TTN_Exon_152C | TTN_Exon_152C | 0.1% | 0.0% |
| 2:179534085-179534189 | 1 | TTN_Exon_152B | TTN_Exon_152B | 0.1% | 0.0% |
| 2:179534304-179534417 | 1 | TTN_Exon_152A | TTN_Exon_152A | 0.1% | 0.0% |
| 2:179535802-179535912 | 1 | TTN_Exon_151 | TTN_Exon_151 | 0.1% | 0.0% |
| 2:179537119-179537223 | 1 | TTN_Exon_149 | TTN_Exon_149 | 0.1% | 0.0% |
| 2:179537347-179537445 | 1 | TTN_Exon_148 | TTN_Exon_148 | 0.1% | 0.0% |
| 2:179539026-179539151 | 1 | TTN_Exon_146 | TTN_Exon_146 | 0.1% | 0.0% |
| 2:179543459-179543572 | 1 | TTN_Exon_140 | TTN_Exon_140 | 0.1% | 0.0% |
| 2:179545791-179545913 | 1 | TTN_Exon_135 | TTN_Exon_135 | 0.1% | 0.0% |
| 2:179547409-179547645 | 1 | TTN_Exon_132 | TTN_Exon_132 | 0.1% | 0.0% |
| 2:179548711-179548824 | 1 | TTN_Exon_130 | TTN_Exon_130 | 0.1% | 0.0% |
| 2:179548712-179548824 | 1 | TTN_Exon_130 | TTN_Exon_130 | 0.1% | 0.0% |
| 2:179549618-179549731 | 1 | TTN_Exon_127 | TTN_Exon_127 | 0.1% | 0.0% |
| 2:179549965-179550072 | 1 | TTN_Exon_126 | TTN_Exon_126 | 0.1% | 0.0% |
| 2:179560060-179560152 | 1 | TTN_Exon_112 | TTN_Exon_112 | 0.1% | 0.0% |
| 2:179654072-179654239 | 1 | TTN_Exon_013 | TTN_Exon_013 | 0.1% | 0.0% |
| 3:46899719-46899777 | 1 | MYL3_Exon_06 | MYL3_Exon_06 | 0.1% | 0.1% |
| 6:112453940-112454107 | 1 | LAMA4_Exon_28 | LAMA4_Exon_28 | 0.1% | 0.0% |
| 6:7574304-7574500 | 1 | DSP_Exon_16 | DSP_Exon_16 | 0.1% | 0.0% |
| 7:151269735-151269810 | 1 | PRKAG2_Exon_09 | PRKAG2_Exon_09 | 0.1% | 0.2% |
| X:153608579-153608742 | 1 | EMD_Exon_04 | EMD_Exon_04 | 0.1% | 0.0% |
| X:153640408-153640566 | 1 | TAZ_Exon_02 | TAZ_Exon_02 | 0.1% | 0.1% |
| X:153641804-153641919 | 1 | TAZ_Exon_04 | TAZ_Exon_04 | 0.1% | 0.0% |
| X:153648356-153648448 | 1 | TAZ_Exon_08 | TAZ_Exon_08 | 0.1% | 0.1% |
| X:153649227-153649358 | 1 | TAZ_Exon_11 | TAZ_Exon_11 | 0.1% | 0.0% |
| 10:21112122-21115483 | 2 | NEBL_Exon_19 | NEBL_Exon_18 | 0.1% | 0.0% |
| 10:21129653-21134312 | 2 | NEBL_Exon_13 | NEBL_Exon_12 | 0.1% | 0.0% |
| 12:22089452-25362860 | 2 | ABCC9_Exon_01 | KRAS_Exon_06 | 0.1% | 0.0% |
| 15:63335014-63336045 | 2 | TPM1_Exon_01 | TPM1_Exon_01A_(2) | 0.1% | 0.1% |
| 18:29100751-29101215 | 2 | DSG2_Exon_04 | DSG2_Exon_05 | 0.1% | 0.0% |
| 19:55668649-55668972 | 2 | TNNI3_Exon_02 | TNNI3_Exon_01 | 0.1% | 0.0% |
| 2:179531536-179534189 | 2 | TTN_Exon_152C | TTN_Exon_152B | 0.1% | 0.0% |
| 2:179534304-179535037 | 2 | TTN_Exon_152A | TTN_Exon_152 | 0.1% | 0.0% |
| 2:179559541-179560152 | 2 | TTN_Exon_113 | TTN_Exon_112 | 0.1% | 0.0% |
| 2:179560577-179561910 | 2 | TTN_Exon_111 | TTN_Exon_110 | 0.1% | 0.0% |
| 2:179569589-179570099 | 2 | TTN_Exon_101 | TTN_Exon_100_ | 0.1% | 0.0% |
| 2:179643941-179644947 | 2 | TTN_Exon_023 | TTN_Exon_022 | 0.1% | 0.0% |
| 7:151267242-151269810 | 2 | PRKAG2_Exon_10 | PRKAG2_Exon_09 | 0.1% | 0.0% |
| X:153607830-153608169 | 2 | EMD_Exon_01 | EMD_Exon_02 | 0.1% | 0.1% |
| X:153608287-153608742 | 2 | EMD_Exon_03 | EMD_Exon_04 | 0.1% | 0.0% |
| X:153609098-153609572 | 2 | EMD_Exon_05 | EMD_Exon_06 | 0.1% | 0.0% |
| X:153609227-153640343 | 2 | EMD_Exon_06 | TAZ_Exon_01 | 0.1% | 0.0% |
| X:153641804-153642542 | 2 | TAZ_Exon_04 | TAZ_Exon_05 | 0.1% | 0.1% |
| 10:21112122-21117578 | 3 | NEBL_Exon_19 | NEBL_Exon_17 | 0.1% | 0.0% |
| 2:179505253-179507059 | 3 | TTN_Exon_170 | TTN_Exon_168 | 0.1% | 0.0% |
| 2:179530089-179534189 | 3 | TTN_Exon_152D | TTN_Exon_152B | 0.1% | 0.0% |
| 2:179549618-179550340 | 3 | TTN_Exon_127 | TTN_Exon_125 | 0.1% | 0.0% |
| 2:179560060-179561910 | 3 | TTN_Exon_112 | TTN_Exon_110 | 0.1% | 0.0% |
| X:119602946-153608169 | 3 | LAMP2_Exon_01 | EMD_Exon_02 | 0.1% | 0.1% |
| 2:179534930-179537223 | 4 | TTN_Exon_152 | TTN_Exon_149 | 0.1% | 0.0% |
| 2:179536683-179538452 | 4 | TTN_Exon_150 | TTN_Exon_147 | 0.1% | 0.0% |
| 2:179559541-179561910 | 4 | TTN_Exon_113 | TTN_Exon_110 | 0.1% | 0.0% |
| X:119602946-153608394 | 4 | LAMP2_Exon_01 | EMD_Exon_03 | 0.1% | 0.0% |
| 2:179534304-179537223 | 5 | TTN_Exon_152A | TTN_Exon_149 | 0.1% | 0.0% |
| 2:179534930-179537445 | 5 | TTN_Exon_152 | TTN_Exon_148 | 0.1% | 0.0% |
| 2:179535802-179538452 | 5 | TTN_Exon_151 | TTN_Exon_147 | 0.1% | 0.0% |
| 2:179547409-179549491 | 5 | TTN_Exon_132 | TTN_Exon_128 | 0.1% | 0.0% |
| 2:179534930-179538452 | 6 | TTN_Exon_152 | TTN_Exon_147 | 0.1% | 0.0% |
| 11:47362539-47367936 | 8 | MYBPC3_Exon_20 | MYBPC3_Exon_12 | 0.1% | 0.0% |
| 2:179534930-179539855 | 8 | TTN_Exon_152 | TTN_Exon_145 | 0.1% | 0.0% |
| 2:179531536-179539151 | 10 | TTN_Exon_152C | TTN_Exon_146 | 0.1% | 0.0% |
| 10:88428434-88492748 | 16 | LDB3_Exon_01 | LDB3_Exon_16 | 0.1% | 0.0% |
| 12:33021846-33022011 | 1 | PKP2_Exon_04 | PKP2_Exon_04 | 0.0% | 6.0% |
| 1:156104963-156105118 | 1 | LMNA_Exon_05 | LMNA_Exon_05 | 0.0% | 0.2% |
| 1:156108856-156108912 | 1 | LMNA_Exon_12 | LMNA_Exon_12 | 0.0% | 0.1% |
| 1:201341140-201341184 | 1 | TNNT2_Exon_04 | TNNT2_Exon_04 | 0.0% | 0.4% |
| 1:201341258-201341298 | 1 | TNNT2_Exon_03 | TNNT2_Exon_03 | 0.0% | 0.1% |
| 1:236900407-236900529 | 1 | ACTN2_Exon_09 | ACTN2_Exon_09 | 0.0% | 0.5% |
| 1:237494163-237494297 | 1 | RYR2_Exon_03 | RYR2_Exon_03 | 0.0% | 0.1% |
| 1:237729852-237730090 | 1 | RYR2_Exon_28 | RYR2_Exon_28 | 0.0% | 0.1% |
| 1:237753925-237754307 | 1 | RYR2_Exon_31 | RYR2_Exon_31 | 0.0% | 0.1% |
| 1:237774047-237774303 | 1 | RYR2_Exon_36 | RYR2_Exon_36 | 0.0% | 0.1% |
| 1:237823270-237823389 | 1 | RYR2_Exon_55 | RYR2_Exon_55 | 0.0% | 0.1% |
| 11:47354730-47354899 | 1 | MYBPC3_Exon_30 | MYBPC3_Exon_30 | 0.0% | 0.1% |
| 11:47357413-47357577 | 1 | MYBPC3_Exon_26 | MYBPC3_Exon_26 | 0.0% | 0.5% |
| 11:47362674-47362810 | 1 | MYBPC3_Exon_19 | MYBPC3_Exon_19 | 0.0% | 0.1% |
| 12:111348866-111348994 | 1 | MYL2_Exon_07 | MYL2_Exon_07 | 0.0% | 0.1% |
| 12:22086701-22086872 | 1 | ABCC9_Exon_02 | ABCC9_Exon_02 | 0.0% | 0.1% |
| 14:23872909-23873002 | 1 | MYH6_Exon_09 | MYH6_Exon_09 | 0.0% | 0.1% |
| 14:23874417-23874603 | 1 | MYH6_Exon_05 | MYH6_Exon_05 | 0.0% | 0.1% |
| 14:23895158-23895305 | 1 | MYH7_Exon_19 | MYH7_Exon_19 | 0.0% | 0.1% |
| 15:63349169-63349332 | 1 | TPM1_Exon_03 | TPM1_Exon_03 | 0.0% | 1.0% |
| 17:37821954-37822377 | 1 | TCAP_Exon_02 | TCAP_Exon_02 | 0.0% | 0.1% |
| 17:39913871-39914051 | 1 | JUP_Exon_11 | JUP_Exon_11 | 0.0% | 0.1% |
| 17:39914636-39914785 | 1 | JUP_Exon_10 | JUP_Exon_10 | 0.0% | 0.1% |
| 18:29122468-29122830 | 1 | DSG2_Exon_14 | DSG2_Exon_14 | 0.0% | 0.1% |
| 18:29178516-29178653 | 1 | TTR_Exon_04 | TTR_Exon_04 | 0.0% | 0.1% |
| 19:55667956-55668027 | 1 | TNNI3_Exon_04 | TNNI3_Exon_04 | 0.0% | 0.4% |
| 2:179452611-179452961 | 1 | TTN_Exon_254 | TTN_Exon_254 | 0.0% | 0.1% |
| 2:179479559-179479710 | 1 | TTN_Exon_209 | TTN_Exon_209 | 0.0% | 0.1% |
| 2:179518379-179518443 | 1 | TTN_Exon_155E | TTN_Exon_155E | 0.0% | 0.4% |
| 2:179518724-179518837 | 1 | TTN_Exon_155C | TTN_Exon_155C | 0.0% | 0.2% |
| 2:179523884-179523997 | 1 | TTN_Exon_154 | TTN_Exon_154 | 0.0% | 0.1% |
| 2:179527678-179527797 | 1 | TTN_Exon_153 | TTN_Exon_153 | 0.0% | 0.6% |
| 20:30411265-30411400 | 1 | MYLK2_Exon_05 | MYLK2_Exon_05 | 0.0% | 0.1% |
| 20:30419792-30419954 | 1 | MYLK2_Exon_12 | MYLK2_Exon_12 | 0.0% | 0.2% |
| 3:14174351-14174450 | 1 | TMEM43_Exon_06 | TMEM43_Exon_06 | 0.0% | 0.1% |
| 3:46902435-46902492 | 1 | MYL3_Exon_02 | MYL3_Exon_02 | 0.0% | 0.1% |
| 6:112457310-112457471 | 1 | LAMA4_Exon_25 | LAMA4_Exon_25 | 0.0% | 0.1% |
| 6:112469344-112469553 | 1 | LAMA4_Exon_18 | LAMA4_Exon_18 | 0.0% | 0.1% |
| 6:112508637-112508818 | 1 | LAMA4_Exon_08 | LAMA4_Exon_08 | 0.0% | 0.1% |
| 6:7562870-7563028 | 1 | DSP_Exon_05 | DSP_Exon_05 | 0.0% | 0.1% |
| 7:151483541-151483642 | 1 | PRKAG2_Exon_02 | PRKAG2_Exon_02 | 0.0% | 0.1% |
| X:100662683-100662906 | 1 | GLA_Exon_01 | GLA_Exon_01 | 0.0% | 0.4% |
| X:119572994-119573163 | 1 | LAMP2_Exon_09B | LAMP2_Exon_09B | 0.0% | 0.1% |
| X:119575570-119575764 | 1 | LAMP2_Exon_08 | LAMP2_Exon_08 | 0.0% | 0.2% |
| X:119582810-119582998 | 1 | LAMP2_Exon_04 | LAMP2_Exon_04 | 0.0% | 0.1% |
| 14:23874294-23874603 | 2 | MYH6_Exon_06 | MYH6_Exon_05 | 0.0% | 4.2% |
| 10:92678607-92679040 | 2 | ANKRD1_Exon_04 | ANKRD1_Exon_03 | 0.0% | 0.1% |
| 11:47371550-47372181 | 2 | MYBPC3_Exon_04 | MYBPC3_Exon_03 | 0.0% | 0.1% |
| 15:63340760-63349332 | 2 | TPM1_Exon_02A_(1) | TPM1_Exon_03 | 0.0% | 0.1% |
| 2:179519157-179522899 | 2 | TTN_Exon_155A | TTN_Exon_155D | 0.0% | 0.1% |
| 2:179523717-179523997 | 2 | TTN_Exon_155 | TTN_Exon_154 | 0.0% | 0.2% |
| X:119590491-119603039 | 2 | LAMP2_Exon_02 | LAMP2_Exon_01 | 0.0% | 0.1% |
| X:119602946-153607941 | 2 | LAMP2_Exon_01 | EMD_Exon_01 | 0.0% | 0.4% |
| X:153608035-153608394 | 2 | EMD_Exon_02 | EMD_Exon_03 | 0.0% | 0.1% |
| 2:179485814-179486750 | 3 | TTN_Exon_195 | TTN_Exon_193 | 0.0% | 0.1% |
| X:100652782-100653949 | 3 | GLA_Exon_07 | GLA_Exon_05 | 0.0% | 0.1% |
| X:153642423-153648100 | 3 | TAZ_Exon_05 | TAZ_Exon_07 | 0.0% | 0.1% |
| 2:179522789-179523997 | 4 | TTN_Exon_155D | TTN_Exon_154 | 0.0% | 0.1% |
| 10:75830718-75843286 | 5 | VCL_Exon_04 | VCL_Exon_08 | 0.0% | 0.1% |
| 2:179519157-179523997 | 5 | TTN_Exon_155A | TTN_Exon_154 | 0.0% | 0.1% |
| X:119576439-119589440 | 5 | LAMP2_Exon_07 | LAMP2_Exon_03 | 0.0% | 0.1% |
| X:119602946-153609177 | 6 | LAMP2_Exon_01 | EMD_Exon_05 | 0.0% | 0.1% |
| X:100655639-119573163 | 7 | GLA_Exon_04 | LAMP2_Exon_09B | 0.0% | 0.1% |
| X:153608579-153641919 | 7 | EMD_Exon_04 | TAZ_Exon_04 | 0.0% | 0.1% |
| 2:179460219-179466306 | 9 | TTN_Exon_244 | TTN_Exon_236 | 0.0% | 0.1% |
| 12:32945343-33049680 | 14 | PKP2_Exon_14 | PKP2_Exon_01 | 0.0% | 0.1% |
| X:119602946-153649358 | 18 | LAMP2_Exon_01 | TAZ_Exon_11 | 0.0% | 0.1% |
| 2:179466714-179485367 | 37 | TTN_Exon_233 | TTN_Exon_197 | 0.0% | 0.1% |

*Please note that these calls may include false positives, they have not all been confirmed by ddPCR.
